# Anti-mGluR5 encephalitis: distinctive clinical features and antibody patterns in the Chinese population

**DOI:** 10.3389/fimmu.2026.1796608

**Published:** 2026-06-15

**Authors:** Tianyu Gao, Xinjing Zhao, Chen Chen, Zhe Wang, Jing Han, Xiaoqi Lin, Yashuang Wu, Xiaolin Yu, Hao Zheng, Xiang Ye, Wei Zhao, Guangrun Xu, Michael D. Geschwind, Guoyu Zhou

**Affiliations:** 1 Department of Neurology, Qilu Hospital of Shandong University, Jinan, China; 2 Department of Geriatric Medicine, Qilu Hospital of Shandong University, Jinan, China; 3 College of Acupuncture and Tuina, Shandong University of Traditional Chinese Medicine, Jinan, China; 4 Acupuncture Department, Affiliated Hospital of Shandong University of Traditional Chinese Medicine, Jinan, China; 5 Department of Neurology, University of California, San Francisco, San Francisco, United States

**Keywords:** anti-mGluR5 encephalitis, autoantibodies, Chinese population, clinical phenotypes, tumor association

## Abstract

**Background:**

Anti-metabotropic glutamate receptor 5 (mGluR5) encephalitis is a rare subtype of autoimmune encephalitis, most often associated with Hodgkin’s lymphoma, and accounts for less than 1% of reported cases worldwide. Current knowledge is primarily based on isolated reports from Western populations, with limited data from Asia. The clinical spectrum, tumor associations, and antibody detection patterns in Chinese patients remain poorly understood.

**Methods:**

We retrospectively reviewed eight consecutive patients with anti-mGluR5 encephalitis diagnosed at a single tertiary referral center between October 2019 and March 2025. Demographic, clinical, neuroimaging, antibody testing, treatment, and outcome data were analyzed. In addition, a systematic review of the Chinese literature identified 31 previously reported cases. Combined institutional and literature cases (n = 39) were summarized and contextualized within the existing international literature.

**Results:**

The age at onset was 42.5 ± 19.7 years (range: 7-78), with a male-to-female ratio of 8:5. Cognitive impairment (61.5%) was the most frequent presenting feature, followed by behavioral disturbances (53.8%) and seizures (53.8%). Tumors were identified in only 7.7% of Chinese patients, notably lower than the 61.5% reported in international literature, with Hodgkin’s lymphoma rarely observed in this population. Serum anti-mGluR5 antibodies were detected in all tested patients, while cerebrospinal fluid positivity was observed in 42.4% of paired samples. First-line immunotherapy (steroids, intravenous immunoglobulin, or plasma exchange) led to clinical improvement in most patients, with 89.5% achieving a favorable outcome (Modified Rankin Scale ≤ 2) at last follow-up. Decreased consciousness at onset was significantly associated with a poorer prognosis (p = 0.009).

**Conclusions:**

Chinese patients with anti-mGluR5 encephalitis exhibit distinctive clinical and immunological features, particularly in terms of tumor associations and antibody detection patterns. These findings expand our understanding of population-specific characteristics in anti-mGluR5 encephalitis and underscore the need for tailored diagnostic strategies and early immunotherapy to optimize outcomes.

## 1 Introduction

Autoimmune encephalitis (AE) encompasses a spectrum of immune‐mediated inflammatory disorders of the central nervous system (CNS), presenting with diverse neuropsychiatric manifestations such as behavioral changes, psychosis, seizures, memory impairments, and altered levels of consciousness (1–3). Over the past two decades, the identification of more than 25 novel neuronal surface and intracellular autoantibodies has substantially improved diagnostic accuracy and broadened our understanding of AE, including rare subtypes such as anti–metabotropic glutamate receptor 5 (mGluR5) encephalitis (4, 5).

Anti-mGluR5 encephalitis, first described in 2011 in association with Ophelia syndrome, is a rare antibody-mediated disorder targeting mGluR5, a G protein–coupled receptor highly expressed in the hippocampus, amygdala, and other limbic structures (6). Clinically, it manifests with heterogeneous features, most commonly cognitive impairment, seizures, and psychiatric disturbances, which can delay diagnosis even in tertiary care settings. In many regions including China, the historical lack of routine mGluR5 antibody testing has limited recognition of this entity.

In recent years, advances in diagnostic techniques have led to increased detection of anti-mGluR5 encephalitis worldwide, including sporadic reports from China. However, systematic characterization of Chinese patients remains scarce. Genetic predispositions, environmental factors, and healthcare system variations may contribute to population-specific clinical features and management patterns. Moreover, methodological differences—particularly between commercial and in-house cell-based assays—complicate direct comparisons between studies.

To address these gaps, we report eight consecutive Chinese patients with anti-mGluR5 encephalitis diagnosed at a single tertiary referral center, alongside a comprehensive review of all previously reported Chinese cases. By pooling institutional and literature data, we aim to delineate the clinical characteristics, tumor associations, antibody detection patterns, treatment responses, and outcomes in this population, and to contextualize these findings within the broader international literature. Our results may help refine diagnostic strategies and inform evidence-based management for this rare but increasingly recognized form of AE.

## 2 Methods

### 2.1 Patients

We retrospectively included all patients diagnosed with anti−mGluR5 encephalitis at the Department of Neurology, Qilu Hospital of Shandong University, a tertiary care center in Eastern China, between October 2019 and March 2025. Diagnosis followed the 2016 clinical approach to autoimmune encephalitis by Graus et al (7)., requiring :(1) subacute onset (< 3 months) of memory deficits, altered mental status, or psychiatric symptoms; (2) new focal CNS findings, seizures not explained by a prior seizure disorder, CSF pleocytosis, or MRI features suggestive of encephalitis; (3) presence of mGluR5 antibodies in serum and/or CSF; and (4) reasonable exclusion of alternative causes.

Patients with coexisting neuronal autoantibodies (e.g., NMDAR, LGI1; full list assessed in this study) were excluded from the primary comprehensive analysis to avoid confounding by overlapping syndromes, their clinical information is provided separately in **Supplementary Materials** for reference.

Demographic data, prodromal and presenting features, ancillary testing (CSF, EEG, MRI), tumor screening, immunotherapy regimens, relapses, and outcomes (mRS at nadir and last follow−up) were extracted from the electronic medical records using a standardized template. Laboratory platforms and antibody testing procedures are described in Section 2.2.

This study adhered to the Declaration of Helsinki and was approved by the Ethics Committee of Qilu Hospital, Shandong University (No. KYLL−202206−026). Written informed consent for participation and publication was obtained from all patients or their legally authorized representatives.

### 2.2 Data collection

For each institutional patient, demographic characteristics, prodromal symptoms, clinical manifestations, MRI and EEG findings, CSF results, tumor status, antibody test site and titer, treatment regimen, relapses, and last Modified Rankin Scale (mRS) scores were recorded using a standardized data extraction form. Laboratory and imaging data were obtained within the first two weeks of admission unless otherwise specified. Tumor screening included chest/abdominal CT or MRI, whole-body PET-CT, and relevant tumor markers; follow-up screening was performed at the discretion of treating physicians.

### 2.3 Antibody detection

Among the eight institutional patients, five underwent antibody testing in both serum and cerebrospinal fluid (CSF), and three had testing performed in serum only. All available samples were screened for a panel of neuronal surface antibodies, including NMDAR, AMPAR (GluR1/2), LGI1, CASPR2, GABA_BR, DPPX, IgLON5, GAD65, mGluR5, and MOG using cell-based assays (CBA) on transfected HEK293 cells (EUROIMMUN, Lübeck, Germany). Serum samples were tested at a dilution of 1:10 and CSF samples at 1:1, according to the manufacturer’s instructions. Antibody positivity was defined based on characteristic immunofluorescence patterns. Antibody titers were semi-quantitatively graded according to fluorescence intensity (e.g., 1:10, 1:32, 1:100, etc.). Positive or equivocal results were confirmed using tissue-based assays (TBA) on monkey cerebellum sections to visualize antibody binding patterns (8–10). All antibody testing (CBA and TBA) was performed at the EUROIMMUN Laboratory, Hangzhou, China. Each assay included positive and negative controls and was conducted under standardized quality control procedures, with participation in external quality assessment programs.

### 2.4 Literature review

A systematic literature search was conducted in PubMed, Embase, Web of Science, and CNKI from database inception to March 31, 2025, using the following terms: (“metabotropic glutamate receptor 5” OR “mGluR5”) AND (“autoimmune encephalitis” OR “encephalitis”). Additional filters included human subjects, case reports or case series, and English or Chinese language. Bibliographies of retrieved articles were screened for additional cases.

Inclusion criteria for literature cases were (1) diagnosis of anti-mGluR5 encephalitis confirmed by CBA or equivalent assay, (2) detailed clinical data available, and (3) absence of duplicate reporting. Exclusion criteria matched those for institutional cases. To avoid duplicate reporting, reports from the same institution were carefully cross-checked by comparing dates of publication and specific patient characteristics. The methodological quality of the included studies was assessed using the Joanna Briggs Institute (JBI) Critical Appraisal Checklists for case reports and case series. Data were extracted from all eligible reports by two authors (TG and XZ). The extracted data included demographics, clinical features, MRI/EEG findings, CSF results, antibody test site and titer, tumor status, treatment, relapses, and outcomes. All disagreements were resolved by discussion between two authors (TG and XZ) with supervision by a third author (GZ). The agreement in selecting case reports between the two authors was excellent, as shown by a Cohen’s κ statistic of 0.90. Clinical manifestations, treatment strategies, and follow−up information of Chinese patients with isolated anti-mGluR5 antibodies (including both institutional and literature cases) are summarized in **Supplementary Tables 1A**, **2A**. Corresponding data for Chinese patients with coexisting neuronal autoantibodies are presented in **Supplementary Tables 1B**, **2B** for reference. Data from patients reported in the international literature are presented in **Supplementary Tables 1C**, **2C**. A detailed flowchart of patient screening and grouping is provided in **Supplementary Figure 1**.

### 2.5 Statistical analysis

Continuous variables were tested for normality using the Shapiro–Wilk test and are presented as mean ± standard deviation (SD) or median with interquartile range (IQR), depending on data distribution. Categorical variables are expressed as counts and percentages. Group comparisons were performed using Student’s t-test or the Mann–Whitney U test for continuous variables and the chi-square or Fisher’s exact test for categorical variables, as appropriate. Univariable analyses were performed to explore factors associated with poor prognosis (mRS > 2 at last follow-up) and relapse. The distribution of major clinical symptoms was compared according to age group, tumor status, and antibody-positive site. Analyses of serum anti-mGluR5 antibody titers were performed in patients with analyzable titer data to assess their associations with initial disease severity, prognosis, and initial clinical symptoms. A two-sided p < 0.05 was considered statistically significant. Statistical analyses were performed using SPSS version 26.0 (IBM Corp., Armonk, NY, USA).

## 3 Results

### 3.1 Participants

A total of 66 patients with anti-mGluR5 encephalitis were identified, comprising 11 diagnosed at Qilu Hospital of Shandong University and 55 reported in the literature (6, 8–33). Among the 66 identified patients, 52 were from Chinese regions. Thirteen of these patients had multiple neuronal antibodies (e.g., NMDAR, LGI1), whereas the remaining 39 patients with isolated mGluR5 antibodies were included in the final analysis, comprising 8 patients from Qilu Hospital and 31 from the published literature. Detailed demographic and baseline characteristics for all included patients are provided in **Table 1**.

**Table 1:** Clinical profiles of anti-mGluR5 encephalitis patients.

Variable	Mean ± SD or n/N(%)
Age	42.5 ± 19.7
Sex	
male	24/39 (61.5%)
female	15/39 (38.5%)
Prodromal symptoms	16/39 (41.0%)
headache	8/39 (20.5%)
fever	11/39 (28.2%)
flu-like	7/39 (17.9%)
weight loss	0/39 (0.0%)
Tumor	3/39 (7.7%)
Key clinical presentations	
mental and behavior disorder	21/39 (53.8%)
altered cognition	24/39 (61.5%)
sleep disturbances	14/39 (35.9%)
seizures	21/39 (53.8%)
dLOC	6/39 (15.4%)
Relapse	5/39 (12.8%)
Initial mRS scores	3.1 ± 1.2
CSF abnormality^a^	24/37 (64.9%)
increased protein concentration	8/15 (53.3%)
leukocytosis	17/37 (45.9%)
OCB positive	7/23 (30.4%)
MRI abnormality^b^	20/39 (51.3%)
EEG abnormality	18/25 (72.0%)
epileptiform discharge	12/18 (66.7%)
Follow-up^c^	
favorable prognosis	34/38 (89.5%)
unfavorable prognosis	4/38 (10.5%)

Data are presented as mean ± SD or n/N (%), where n denotes the number of patients with positive findings, and N denotes the total number of patients or those with available data (sample size may vary due to missing information).

^a^ CSF reference ranges: WBC ≤ 5/mm³; protein, 15–45 mg/dL; OCB, negative.

^b^ MRI abnormalities include hyperintense signals on T2-weighted and fluid-attenuated inversion recovery (FLAIR) images and/or subtle restricted diffusion on diffusion-weighted imaging (DWI).

^c^ Favorable prognosis: mRS ≤ 2; unfavorable prognosis: mRS > 2.

dLOC, decreased level of consciousness; mRS, Modified Rankin Scale; CSF, cerebrospinal fluid; OCB, oligoclonal bands in CSF; MRI, magnetic resonance imaging; EEG, electroencephalography.

### 3.2 Clinical presentations

**Figure 1:**
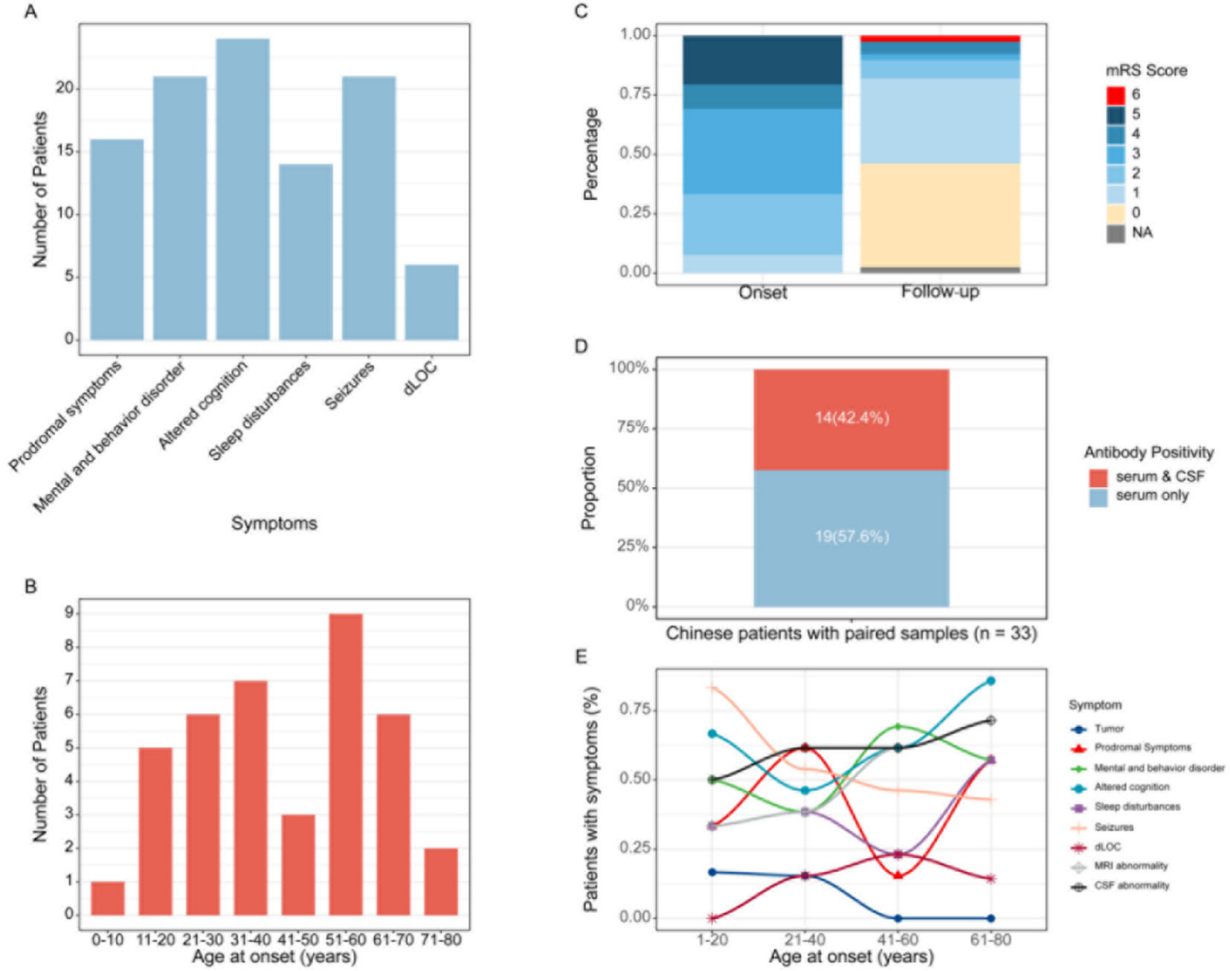
Age distribution, initial clinical features, functional outcomes, and antibody profiles in patients with anti-mGluR5 encephalitis. **(A)** Distribution of patients by initial clinical symptoms. Altered cognition was the most common presentation, encompassing a spectrum from psychiatric and behavioral disturbances to decreased level of consciousness (dLOC). **(B)** Age distribution at disease onset. The highest prevalence was observed in patients aged 51–60 years, accounting for 9 cases (23.1%). **(C)** Modified Rankin Scale (mRS) scores at disease onset and at last follow-up. Functional status improved in most patients after treatment. **(D)** Antibody detection in paired serum and cerebrospinal fluid (CSF) samples (n = 33). All patients tested positive for mGluR5 antibodies in serum; 19 (57.6%) exhibited serum-only positivity, whereas 14 (42.4%) had antibodies detectable in both serum and CSF. **(E)** Age-stratified cumulative clinical symptom profiles. In patients aged ≤ 20 years (n = 6), seizures were most frequent (83.3%, 5/6), with a declining incidence in older age groups. dLOC, decreased level of consciousness; mRS, Modified Rankin Scale; CSF, cerebrospinal fluid; MRI, magnetic resonance imaging.

The mean age at onset among the 39 Chinese patients was 42.5 ± 19.7 years, with a male predominance (61.5%, 24/39). Neurological manifestations were dominated by cognitive impairment, the most prevalent symptom in 61.5% (24/39) of patients (**Figure 1A**). This was followed by mental and behavioral disturbances (53.8%, 21/39) and seizures (53.8%, 21/39). Sleep disturbances occurred in 35.9% (14/39) of patients, while decreased level of consciousness (dLOC) was observed in 15.4% (6/39) of cases. The age distribution showed that patients aged 51–60 years had the highest prevalence, accounting for 23.1% of cases (**Figure 1B**). Age-related symptom patterns revealed distinct clinical phenotypes. In pediatric patients aged ≤ 20 years (n=6), seizures were the most frequent presentation (83.3%, 5/6), with incidence decreasing with advancing age (**Figure 1E**). Prodromal symptoms occurred in 41.0% (16/39) of patients, most frequently fever (68.8%, 11/16) and headache (50.0%, 8/16).

Detailed clinical characteristics and outcomes are summarized in **Table 1**. At follow-up (median: 16.5 months), 89.5% (34/38) of patients achieved favorable outcomes (mRS ≤ 2), with functional improvement from initial mRS scores of 3.1 ± 1.2 (**Figure 1C**). Comparisons stratified by age, tumor status, and antibody-positive site showed no statistically significant differences in the distribution of initial clinical symptoms (**Supplementary Table 3**).

These findings highlight the heterogeneous but overlapping symptom spectrum of anti-mGluR5 encephalitis in Chinese patients, underscoring the need for broad clinical suspicion in individuals presenting with limbic or neuropsychiatric symptoms.

### 3.3 Imaging and EEG findings

**Figure 2:**
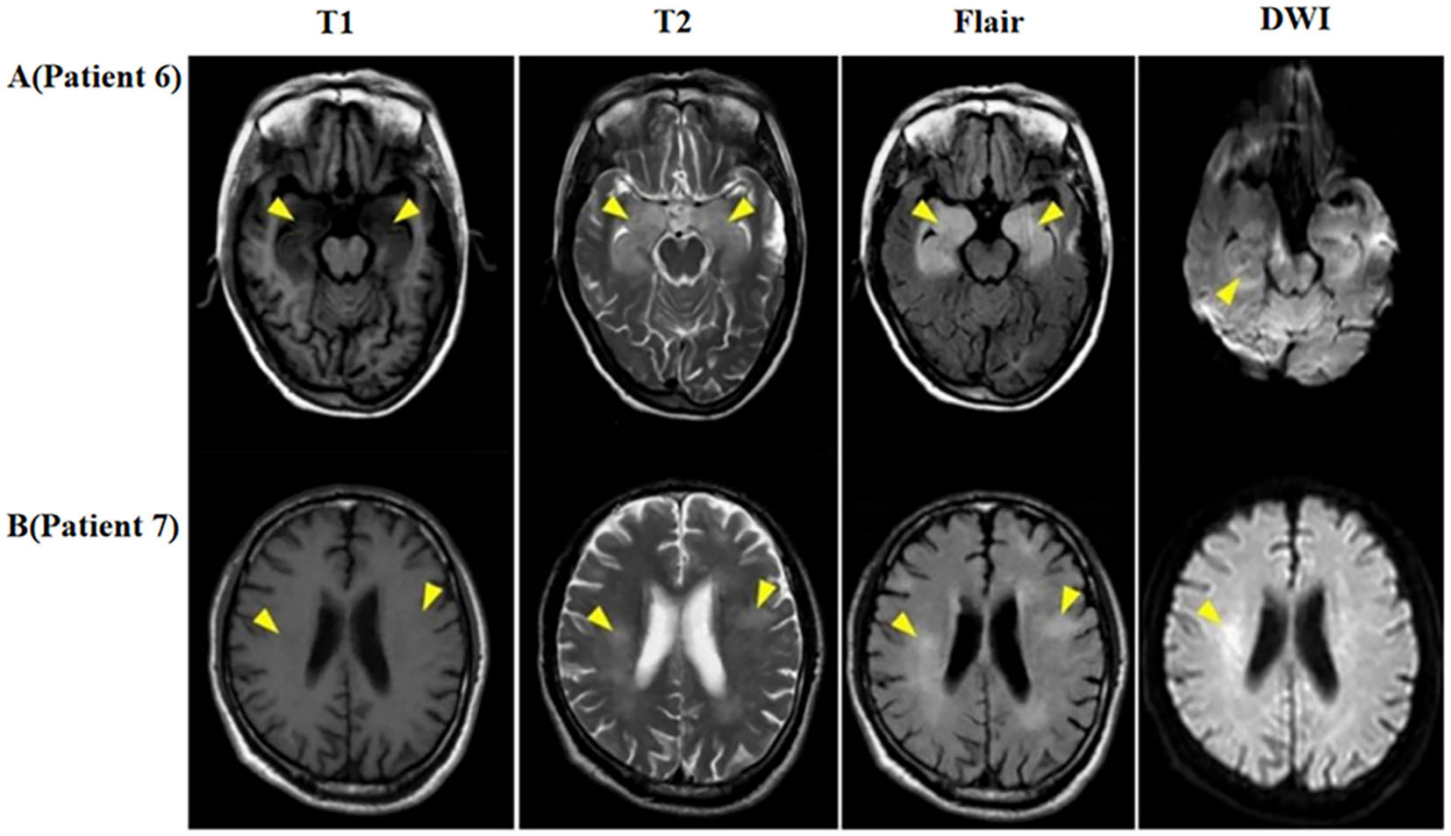
Axial brain MRI findings in two patients with anti-mGluR5 encephalitis. **(A)** Patient 6: Bilateral hippocampal lesions showing hypointensity on T1-weighted images, hyperintensity on T2-weighted and fluid-attenuated inversion recovery (FLAIR) sequences, and subtle restricted diffusion on diffusion-weighted imaging (DWI) (yellow arrowheads). **(B)** Patient 7: Bilateral white matter lesions displaying hypointensity on T1-weighted images, hyperintensity on T2-weighted/FLAIR sequences, and subtle restricted diffusion on DWI (yellow arrowheads). MRI, magnetic resonance imaging; FLAIR, fluid-attenuated inversion recovery; DWI, diffusion-weighted imaging.

Approximately half of the patients (51.3%, 20/39) exhibited MRI abnormalities, most frequently involving the medial temporal lobes (30.0%, 6/20) and hippocampus (25.0%, 5/20). These regions are consistent with predominant limbic system involvement. Representative MRI findings from two patients demonstrating bilateral hippocampal and white matter lesions are shown in **Figure 2**. EEG abnormalities were present in 72.0% (18/25) of patients, with epileptiform discharges detected in 66.7% (12/18) of these cases. These findings support the clinical observation that seizures are a common manifestation in this cohort.

### 3.4 Laboratory findings

#### 3.4.1 Serum and CSF antibody detection patterns

**Figure 3:**
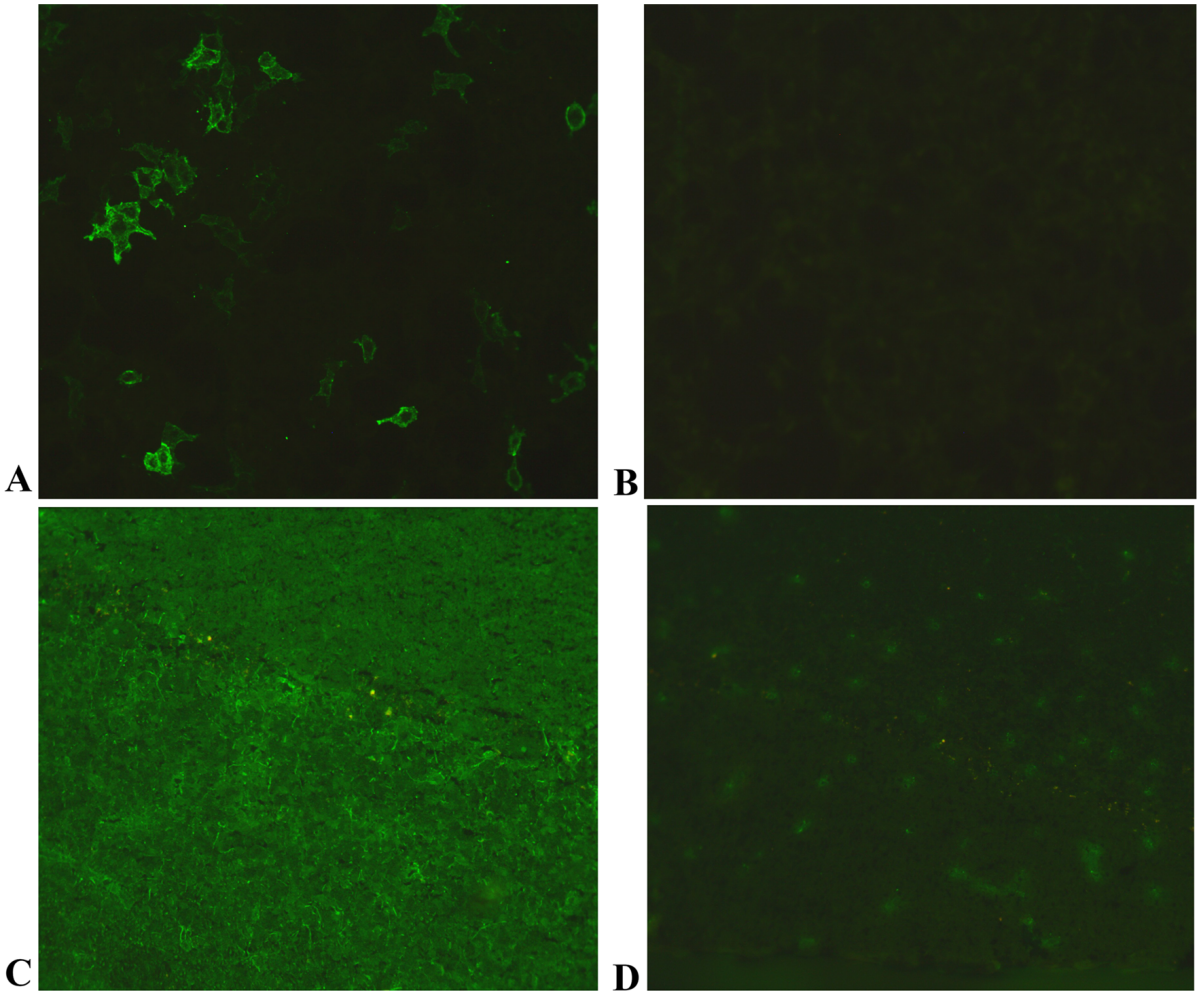
Detection of anti-mGluR5 antibodies. Anti-mGluR5 antibodies were first detected by a cell-based assay (CBA) using HEK293 cells transfected to overexpress human mGluR5, and subsequently confirmed by a tissue-based assay (TBA) on monkey cerebellar sections. **(A)** Serum from patient 5 showed strong cell surface staining of mGluR5-expressing HEK293 cells in the CBA (×200; titer 1:32). **(B)** CSF from patient 5 yielded a negative CBA result, with no observable staining in mGluR5-expressing HEK293 cells (×200). **(C)** The same serum also produced positive staining in the TBA, with prominent neuropil immunoreactivity in the molecular layer of the monkey cerebellum (×100). **(D)** CSF from patient 5 was negative in the TBA, showing no significant neuropil staining in monkey cerebellar tissue (×100). CBA, cell-based assay; TBA, tissue-based assay; CSF, cerebrospinal fluid.

Among the 39 patients, 38 underwent serum antibody testing, and 34 underwent CSF antibody testing; 33 patients had both serum and CSF samples available for paired analysis. Serum anti-mGluR5 antibodies were detected in all patients with paired samples (100%, 33/33), whereas CSF positivity was significantly lower (42.4%, 14/33), yielding an approximate serum-to-CSF detection ratio of 2:1. Representative examples of antibody detection using both cell-based assay (CBA) and tissue-based assay (TBA) are illustrated in (**Figure 3**), demonstrating the differential sensitivity between serum and CSF samples. International literature reports document higher serum antibody titers (≥1:100) in some populations (6/6 vs. 7/24, p = 0.003), and higher rates of CSF antibody positivity (9/10 vs. 15/34, p = 0.013) than observed in our pooled Chinese dataset.

In exploratory analyses, serum anti-mGluR5 antibody titers were not associated with disease severity, prognosis, or initial clinical symptoms (**Supplementary Tables 3, 4**).

#### 3.4.2 Additional CSF findings

CSF abnormalities were observed in 64.9% (24/37) of patients, including leukocytosis (45.9%, 17/37), positive oligoclonal bands (30.4%, 7/23), and elevated protein levels (53.3%, 8/15). These findings indicate a frequent but variable inflammatory response within the CNS in anti-mGluR5 encephalitis.

### 3.5 Tumor association

**Figure 4:**
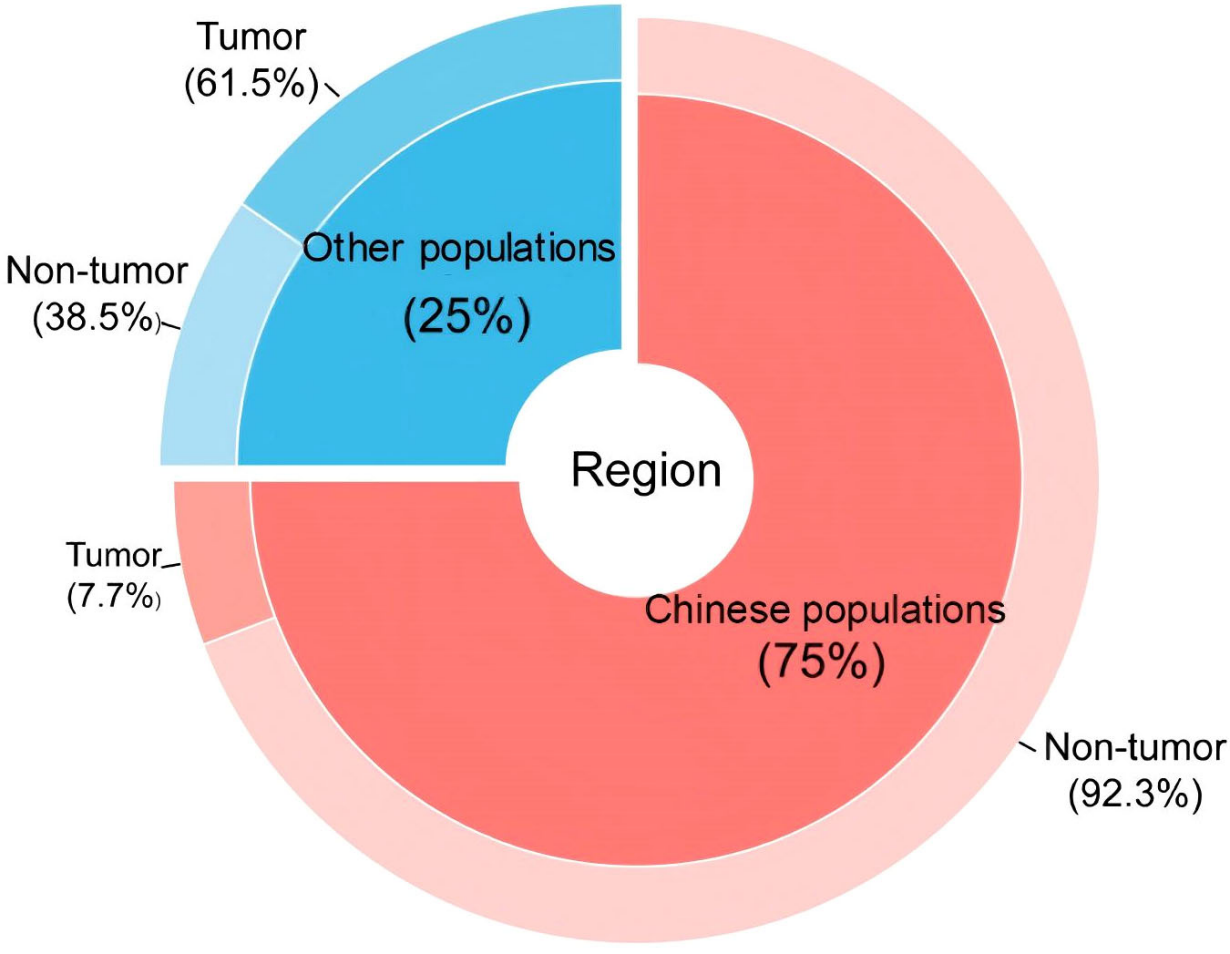
Tumor prevalence patterns in anti-mGluR5 encephalitis across populations. Donut chart illustrating the proportion of patients from Chinese populations (75.0%) and international literature (25.0%), stratified by tumor status. Tumor prevalence was notably low in Chinese patients (7.7%) while international reports document higher rates (61.5%), with the majority of Chinese patients being tumor-free (92.3%) compared to 38.5% in international reports.

Associated tumors were identified in 3 (7.7%) Chinese patients, comprising pulmonary adenocarcinoma, intramedullary spinal teratoma, and gangliocytoma. International literature reports document paraneoplastic cases in 61.5% of patients (8/13; p < 0.001) (**Figure 4**). Of these, 87.5% (7/8) were Hodgkin’s lymphoma, consistent with the well-established association between anti-mGluR5 antibodies and Ophelia syndrome. Given these population-specific patterns, early and comprehensive tumor screening remains recommended, particularly with attention to regional epidemiological factors.

### 3.6 Treatment and outcomes

Immunotherapy was administered to 87.2% (34/39) of patients, with the most common regimens being intravenous immunoglobulin (IVIg) combined with corticosteroids (50.0%, 17/34), corticosteroids alone (32.4%, 11/34), and IVIg alone (17.6%, 6/34). At the last follow-up (median: 16.5 months), mRS scores were available for 38 patients: 34 (89.5%) achieved favorable outcomes (mRS ≤ 2) and 4 (10.5%) had unfavorable outcomes (mRS > 2). Univariable analyses showed that initial dLOC was significantly associated with poor prognosis (p = 0.009), while no clinical factors were significantly associated with relapse, relapsed patients tended to be younger (p = 0.051) (**Table 2**). These outcome data suggest that most patients respond well to first-line immunotherapy, but early identification and treatment remain crucial for preventing long-term disability. Prognosis did not differ significantly among the different immunotherapy regimens (**Supplementary Table 5**). Individual follow-up trajectories are shown in **Supplementary Figure 2**.

**Table 2:** Univariable comparisons of clinical variables according to prognosis and relapse status in patients with anti-mGluR5 encephalitis. .

Variables	Favorable prognosis	Unfavorable prognosis	P value	Non-relapsed	Relapse	P value
Age (years)	40.3 ± 19.7	60.0 ± 12.5	0.060	44.9 ± 19.2	26.6 ± 16.4	0.051
Sex			1.000			0.631
Female	14/34 (41.2%)	1/4 (25.0%)		14/34 (41.2%)	1/5 (20.0%)	
Male	20/34 (58.8%)	3/4 (75.0%)		20/34 (58.8%)	4/5 (80.0%)	
Prodromal symptoms	13/34 (38.2%)	3/4 (75.0%)	0.291	13/34 (38.2%)	3/5 (60.0%)	0.631
Tumor	3/34 (8.8%)	0/4 (0.0%)	1.000	2/34 (5.9%)	1/5 (20.0%)	0.345
Mental and behavior disorder	17/34 (50.0%)	4/4 (100.0%)	0.113	18/34 (52.9%)	3/5 (60.0%)	1.000
Altered cognition	20/34 (58.8%)	3/4 (75.0%)	1.000	21/34 (61.8%)	3/5 (60.0%)	1.000
Sleep disturbances	13/34 (38.2%)	1/4 (25.0%)	1.000	12/34 (35.3%)	2/5 (40.0%)	1.000
Seizures	18/34 (52.9%)	2/4 (50.0%)	1.000	18/34 (52.9%)	3/5 (60.0%)	1.000
dLOC	3/34 (8.8%)	3/4 (75.0%)	0.009**	5/34 (14.7%)	1/5 (20.0%)	1.000
CSF abnormality	21/34 (65.6%)	3/4 (75.0%)	1.000	19/34 (59.4%)	5/5 (100.0%)	0.140
MRI abnormality	16/34 (47.1%)	3/4 (75.0%)	0.604	17/34 (50.0%)	3/5 (60.0%)	1.000
EEG abnormality	15/22 (68.2%)	3/3 (100.0%)	0.534	15/20 (75.0%)	3/5 (60.0%)	0.597
Onset to immunotherapy interval (weeks)	10.1 ± 14.0	4.5 ± 3.5	0.591	10.3 ± 14.3	4.7 ± 4.7	0.512

Data are presented as mean ± SD or n/N (%). Sample size may vary because of missing data. P values were calculated using Student’s t-test or the Mann–Whitney U test for continuous variables and the chi-square test or Fisher’s exact test for categorical variables, as appropriate. Favorable prognosis was defined as mRS ≤ 2 at last follow-up, and unfavorable prognosis as mRS > 2. P value < 0.05 was considered statistically significant. ** P < 0.01.

dLOC, decreased level of consciousness; mRS, Modified Rankin Scale; CSF, cerebrospinal fluid; MRI, magnetic resonance imaging; EEG, electroencephalography.

## 4 Discussion

This study represents the most comprehensive characterization of anti-mGluR5 encephalitis in a Chinese population to date, integrating 8 original cases from our center with 31 additional cases systematically identified from the literature. Our analysis reveals several distinctive features that expand current understanding of this condition: (1) a notably low tumor prevalence, particularly the rare occurrence of Hodgkin’s lymphoma; (2) a substantially higher detection rate of anti-mGluR5 antibodies in serum than in CSF, with a serum-to-CSF detection ratio of approximately 2:1; and (3) the identification of dLOC as a significant predictor of poor functional outcome. Most patients achieved favorable outcomes following first-line immunotherapy, consistent with earlier reports (4, 6, 8, 18). However, the prognostic value of specific clinical variables—such as dLOC—has not been systematically documented in this rare entity.

These findings reveal distinctive epidemiological and immunological characteristics in Chinese patients with anti-mGluR5 encephalitis, which may reflect genetic background, environmental exposures, healthcare accessibility patterns, and diagnostic methodological factors. In the following sections, we discuss these observations in detail, focusing on their potential pathophysiological mechanisms, diagnostic relevance, and therapeutic implications.

### 4.1 Cognitive profile and pathogenic mechanisms

In our cohort, cognitive impairment was the most prevalent neurological manifestation, aligning with the central biological role of mGluR5 in synaptic plasticity and higher cognitive functions. mGluR5 is integral to long-term depression (LTD) and modulates rapid synaptic transmission in the hippocampus (34), while also regulating long-term potentiation (LTP) through interactions with NMDA receptors (35)—both processes being crucial for learning and memory, which are frequently impaired in anti-mGluR5 encephalitis. Animal studies further corroborate these mechanisms: mice infused with patients’ IgG developed memory deficits and heightened anxiety, which reversed following antibody clearance (36).

Given these findings, it is plausible that mGluR5 autoantibodies disrupt glutamatergic signaling within limbic structures, thereby producing the cognitive and behavioral disturbances observed clinically. This is consistent with prior neuroimaging and electrophysiological studies in AE, which have shown functional network disruptions in the hippocampus, amygdala, and related cortical regions (34–36). Such pathophysiological insights emphasize the need for early recognition and intervention to preserve cognitive function in affected patients.

### 4.2 Tumor prevalence and cohort differences

One of the most striking findings in our study is the notably low tumor prevalence in this Chinese population with anti-mGluR5 encephalitis, only 7.7% in our comprehensive dataset, while international literature reports document 61.5% tumor prevalence, the majority of which were Hodgkin’s lymphoma (87.5%) (6, 8, 18). Notably, no Chinese patient in our series had Hodgkin’s lymphoma, despite its strong and well-documented association with anti-mGluR5 antibodies in Western populations.

Several factors may contribute to this distinct pattern. First, genetic background could influence tumor susceptibility. HLA frequencies vary substantially across geographic populations, and specific HLA alleles have been implicated in the development of paraneoplastic syndromes and their associated autoantibody responses (37–39); differences in allele frequency between ethnic groups may partly account for the absence of Hodgkin’s lymphoma in Chinese patients. Second, environmental and epidemiological factors—such as geographic variation in lymphoma incidence—could contribute to this population-specific pattern. Epidemiological studies have shown that Hodgkin’s lymphoma incidence is generally lower in China and other Asian regions than in Europe and North America (40, 41). Third, differences in tumor screening strategies may have affected detection rates: while many Western reports utilized PET-CT or whole-body MRI for tumor surveillance, Chinese cases more commonly underwent standard imaging (e.g., abdominal ultrasound, or routine CT), which may be less sensitive for detecting small or early-stage malignancies.

Although anti-mGluR5 antibodies are classified as intermediate-risk markers for paraneoplastic syndromes (37, 42), our findings suggest that tumor associations may be far less common in the Chinese population than previously assumed. This raises an important question: how can tumor screening protocols for anti-mGluR5 encephalitis be optimized to capture rare but clinically significant neoplasms across different populations? Until further data are available, we recommend adopting high-sensitivity screening—particularly at diagnosis and during follow-up — regardless of ethnic background, to ensure timely detection of associated malignancies.

### 4.3 Diagnostic variability in antibody detection

Our data reveal a clear disparity between serum and CSF anti-mGluR5 antibody detection rates in Chinese patients: in paired samples, serum testing was positive in 100% of cases, whereas CSF testing was positive in only 42.4%, yielding a serum-to-CSF detection ratio of approximately 2:1. This observation aligns with established patterns in autoimmune encephalitis, where antibody distribution between serum and CSF varies by disease subtype (43). For example, NMDAR antibodies are typically more readily detected in CSF, whereas LGI1 and CASPR2 antibodies are more prevalent in serum. Our findings suggest that anti-mGluR5 encephalitis may follow the latter pattern.

Notably, international reports document higher serum antibody titers (≥1:100) in some populations (6/6 vs. 7/24, p = 0.003), alongside higher rates of CSF antibody positivity (90.0% vs. 44.1%, p = 0.013) than observed in our Chinese dataset. This observation may reflect population-specific biological characteristics—for instance, lower antibody titers could reduce detectability in CSF, where antibody concentrations often correlate with disease severity and intrathecal immune activity. Alternatively, methodological factors may contribute: our analysis utilized commercial cell-based assays, while some international studies have employed in-house assays, which can differ in sensitivity and specificity. The semi-quantitative nature of immunofluorescence-based assays and inter-laboratory variability may partly account for differences in antibody detection patterns across studies.

From a clinical perspective, these findings underscore the diagnostic value of serum testing in anti-mGluR5 encephalitis, particularly in settings where CSF results are negative but clinical suspicion remains high. Nevertheless, paired serum and CSF testing remains advisable, as discordant results may carry diagnostic or prognostic implications. Future studies should aim to standardize assay methodologies across centers, enabling more accurate comparisons and potentially uncovering population-specific differences in antibody kinetics and compartmentalization.

### 4.4 Pediatric vs. adult presentations

Age appeared to influence both the clinical phenotype and symptom predominance in Chinese patients with anti-mGluR5 encephalitis. Pediatric patients (≤ 20 years) presented most frequently with seizures (83.3%), a finding consistent with prior reports on pediatric autoimmune encephalitis (44, 45). This heightened seizure susceptibility may relate to the greater excitability and synaptic plasticity of the developing brain, together with the high expression of mGluR5 in the hippocampus and related limbic structures (46, 47). Given the role of mGluR5 in modulating glutamatergic transmission, excessive or aberrant receptor activation in these regions could facilitate epileptogenesis in younger individuals.

In contrast, adult patients were more likely to present with cognitive impairment and behavioral disturbances—symptoms that align with dysfunction of the hippocampus, prefrontal cortex, and other limbic regions, where mGluR5 contributes to hippocampal long-term potentiation as well as prefrontal cortical excitability and persistent activity (34, 35, 48, 49). These age-related differences in symptom profile may reflect developmental neurobiology, differences in immune response, and possibly the pattern of antibody-mediated neuronal network disruption.

From a clinical standpoint, these findings suggest that age-specific diagnostic considerations are warranted. In pediatric patients, new-onset seizures—particularly when accompanied by psychiatric or cognitive symptoms — should prompt early screening for anti-mGluR5 antibodies, even in the absence of overt MRI abnormalities. In adults, subtle or subacute cognitive and behavioral changes should raise suspicion for limbic system–predominant involvement. Tailoring therapeutic strategies to these age-dependent phenotypes could optimize early intervention and improve long-term outcomes.

### 4.5 Prognostic factors

In this cohort, initial dLOC was significantly associated with poor prognosis (p = 0.009), indicating greater residual disability. This observation is consistent with previous studies in autoimmune encephalitis, where altered consciousness correlated with worse cognitive outcomes and poorer functional performance (50, 51).

The association between dLOC and unfavorable prognosis may reflect extensive limbic and extra-limbic involvement, potentially indicating higher antibody titers or more diffuse CNS inflammation at disease onset. Pathophysiologically, widespread disruption of neuronal networks — particularly within the ascending reticular activating system and bilateral limbic structures — could impair consciousness and signal a more aggressive disease trajectory.

From a clinical perspective, early recognition and management of dLOC should be prioritized. This includes prompt initiation of first-line immunotherapy, aggressive investigation for concurrent complications (e.g., seizures, status epilepticus, or metabolic disturbances), and continuous neurological monitoring. In our series, most patients without dLOC responded well to early treatment and achieved favorable outcomes (mRS ≤ 2), underscoring the importance of initial neurological status as a prognostic marker. Future prospective studies should incorporate standardized consciousness assessments at presentation and during the acute phase to further validate dLOC as a robust prognostic indicator across different AE subtypes, including anti-mGluR5 encephalitis.

In summary, our findings expand the current understanding of anti-mGluR5 encephalitis in the Chinese population, revealing a unique clinical profile characterized by lower tumor prevalence, predominant serum antibody positivity, and age-related symptom patterns. The absence of Hodgkin’s lymphoma and distinct serological trends suggest possible genetic, environmental, and methodological influences that warrant further investigation. Early identification—particularly of patients presenting with decreased consciousness — and prompt immunotherapy are crucial for optimizing outcomes. These findings have several potential clinical implications. First, tumor screening should be emphasized at diagnosis in patients with anti-mGluR5 encephalitis, and more sensitive screening strategies may be considered in selected patients. Second, in patients with negative CSF results but high clinical suspicion, repeat antibody testing should be considered, though this approach should be weighed against availability and economic costs. Third, our findings support the effectiveness of early immunotherapy, and future larger studies may further clarify whether initiation within 1 week of onset provides additional prognostic benefit. Future research should prioritize standardized, high-sensitivity tumor screening, uniform antibody testing protocols, and prospective multicenter follow-up to clarify prognostic factors and guide tailored, population-specific management strategies for this rare autoimmune encephalitis.

### 4.6 Study limitations

This study has several limitations. First, the retrospective design and reliance on published case reports and small case series may have introduced selection and publication bias. Second, cognitive impairment and mental/behavioral abnormalities were not standardized in some included cases and were assessed clinically rather than formally, which may have introduced heterogeneity and reduced comparability. Third, methodological variations in antibody detection — particularly the use of commercial kits in our analysis versus in-house assays in some international studies—may have influenced positivity rates and titer comparisons. In addition, because some antibody titers were not reported as specific dilution titers, only a subset of patients could be included in the titer analysis, limiting sample size and comparability. Fourth, variations in tumor screening approaches across studies, with some international reports utilizing PET-CT or whole-body MRI while our cases more commonly underwent standard imaging, could have influenced tumor detection rates. Fifth, the relatively short follow-up in some patients may have led to underestimation of relapse rates and long-term outcomes and may also have limited the detection of occult malignancies. In addition, the small numbers of poor-outcome and relapse events limited the statistical power of the risk-factor analyses, these results should be interpreted cautiously. Finally, the small number of pediatric patients limited the robustness of age-stratified analyses, and conclusions regarding pediatric phenotypes require validation in larger cohorts. Future research should address these limitations through prospective, multicenter studies with standardized diagnostic protocols, high-sensitivity tumor surveillance, and extended follow-up to better assess disease course, treatment response, and relapse risk.

## 5 Conclusions

This study, the largest to date on anti-mGluR5 encephalitis in Chinese patients, reveals distinctive clinical, serological, and oncological characteristics that expand our understanding of this rare condition, notably a low tumor prevalence and rare occurrence of Hodgkin’s lymphoma, reflecting potential population-specific genetic, environmental, or methodological factors. Serum testing demonstrated a substantially higher detection rate than CSF testing, reinforcing its diagnostic value, while an initial decreased level of consciousness was strongly associated with poor functional outcomes. These findings contribute valuable insights for standardized diagnostic protocols with high-sensitivity tumor screening, routine serum antibody testing, and early prognostic assessment; prospective multicenter studies with long-term follow-up are warranted to validate these results and inform evidence-based management strategies for this rare but increasingly recognized autoimmune encephalitis.

## Data Availability

The original contributions presented in the study are included in the article/**Supplementary Material**, further inquiries can be directed to the corresponding author/s.
