# Supplementary figures and images for: Anti-mGluR5 encephalitis: distinctive clinical features and antibody patterns in the Chinese population
Source: Front Immunol. 2026 Jun 15;17:1796608. doi: 10.3389/fimmu.2026.1796608 (PMC13310686; doi:10.3389/fimmu.2026.1796608)

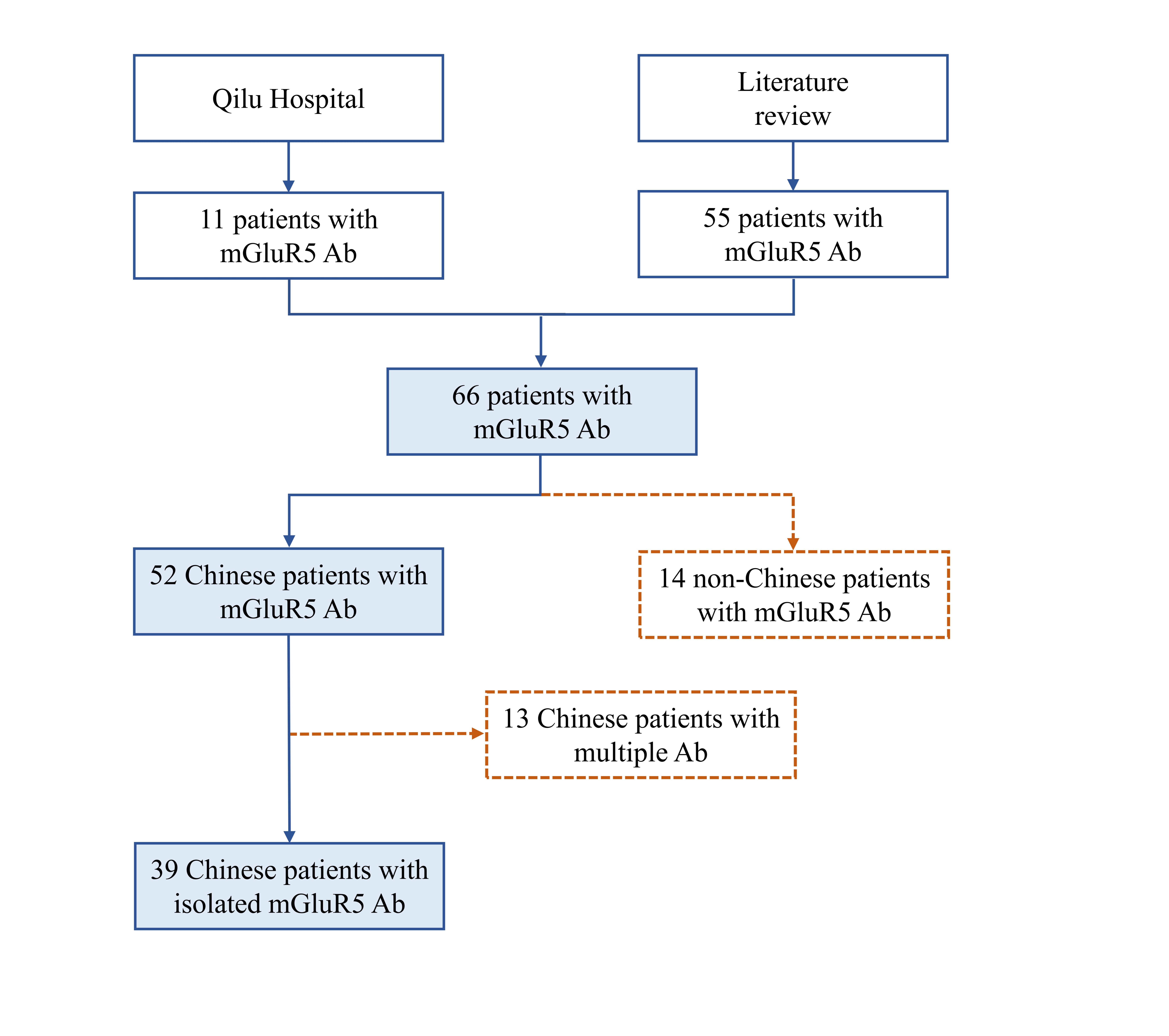

Supplement: Supplementary file 1 [file Image1.tif]

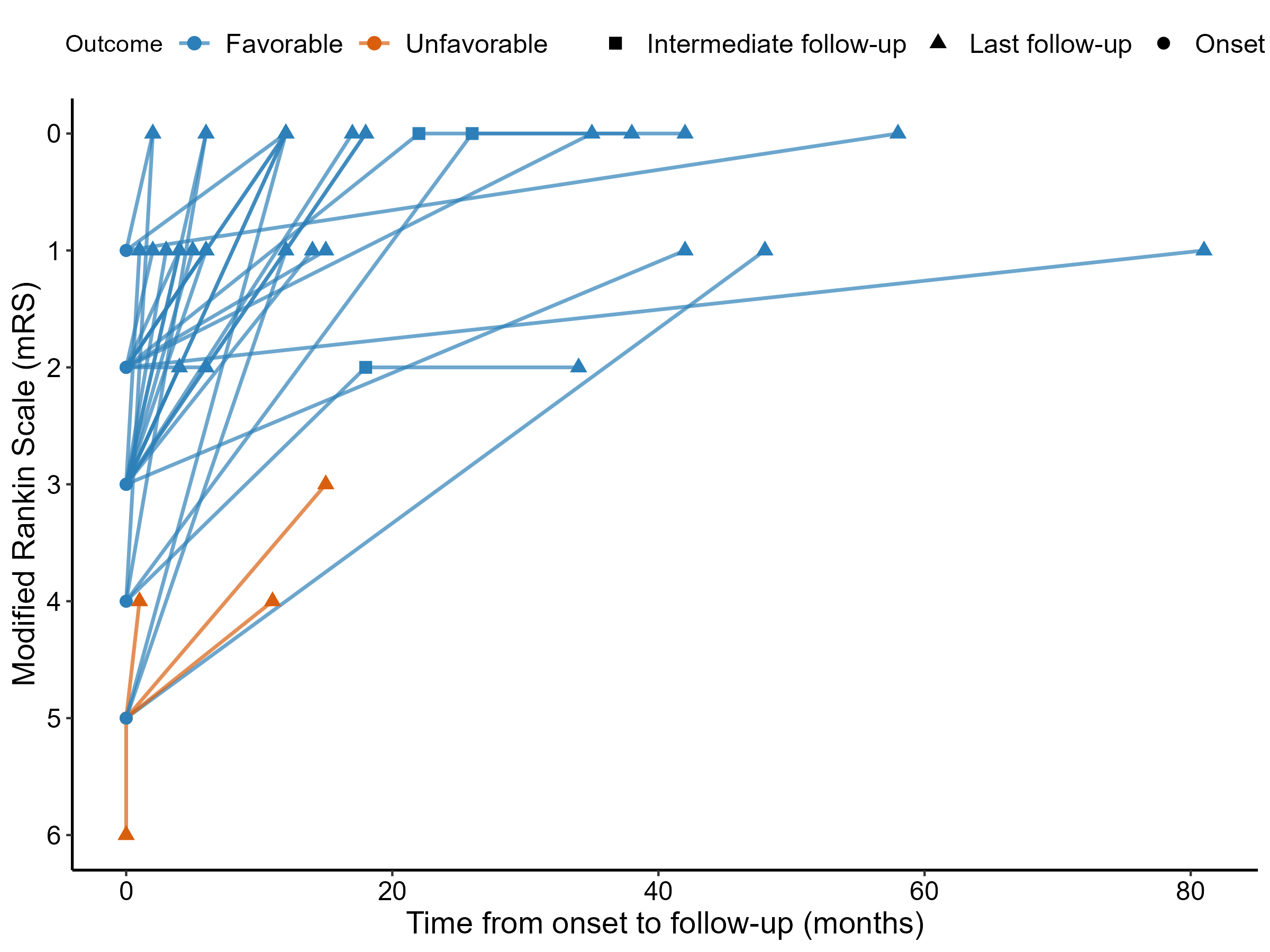

Supplement: Supplementary file 2 [file Image2.tif]
