# Supplementary material for: Anti-mGluR5 encephalitis: distinctive clinical features and antibody patterns in the Chinese population
Source: Front Immunol. 2026 Jun 15;17:1796608. doi: 10.3389/fimmu.2026.1796608 (PMC13310686; doi:10.3389/fimmu.2026.1796608)
Supplement: Supplementary file 3 [file Table1.docx]

**Supplemental material**

**Table S1. Clinical manifestations of patients with anti-mGluR5 encephalitis**

[**Table S1A. Chinese patients with isolated anti-mGluR5 antibodies (n=39)**](#OLE_LINK1)

[**Table S1B. Chinese patients with multiple antibodies (n=13)**](#OLE_LINK2)

[**Table S1C. Non-Chinese patients (n=14)**](#OLE_LINK3)

**Table S2. Treatment strategies and clinical outcomes**

[**Table S2A. Chinese patients with isolated anti-mGluR5 antibodies (n=39)**](#OLE_LINK4)

[**Table S2B. Chinese patients with multiple antibodies (n=13)**](#OLE_LINK5)

[**Table S2C. Non-Chinese patients (n=14)**](#OLE_LINK6)

[**Table S3. Associations of initial clinical symptoms with age, tumor status, antibody-positive site and serum antibody titer in Chinese patients with isolated anti-mGluR5 antibodies**](#OLE_LINK7)

[**Table S4. Association of serum anti-mGluR5 antibody titer with disease severity and prognosis in Chinese patients with isolated anti-mGluR5 antibodies (n=24)**](#OLE_LINK8)

[**Table S5. Comparison of treatment regimens and outcomes in Chinese patients with isolated anti-mGluR5 antibodies (n=33)**](#OLE_LINK12)

**Table S6. Quality assessment of included studies**

[**Table S6A. Quality assessment for the included case report studies**](#OLE_LINK15)

[**Table S6B. Quality assessment for the included case series studies**](#OLE_LINK16)

[**Supplemental Figure S1. Screening and grouping process for patients with mGluR5 antibodies**](#OLE_LINK17)

[**Supplemental Figure S2. Longitudinal follow-up trajectories of mRS scores in Chinese patients with isolated anti-mGluR5 encephalitis.**](#OLE_LINK2)

**Table S1. Clinical manifestations of patients with anti-mGluR5 encephalitis**

**Table S1A. Chinese patients with isolated anti-mGluR5 antibodies (n=39)**

| ID  Sex  Age | Prodromal  Symptoms | Tumor | Tumor Screening  Strategies | Main clinical features | AEDs | CSF Findings | EEG | MRI | Ab Titer |
| --- | --- | --- | --- | --- | --- | --- | --- | --- | --- |
| 1  M  31 | Fever,  headache, dizziness, fatigue | Teratoma | Chest, abdominal  and pelvis CT,  serum tumor  markers, | Headache,  dizziness,  gait instability,  vomiting,  sweating,  generalized tonic-clonic seizure;  developed right  abducent nerve paralysis 1 week later. | Valproate | NA | ED | Normal | S: 1:10  CSF: NA |
| 2  M  57 | None | None | Chest, abdominal  and pelvis CT | Hallucinations, psychosis;  developed gait instability and disorganized speech after 5 days. |  | WBC: 7  Protein: 54mg/dL  IgG: 30.8mg/L  OCB: Neg | ED in frontal central temporal regions | Normal | S: 1:10  CSF: Neg |
| 3  F  24 | None | None | Chest, abdominal  and pelvis CT,  PET-CT | Hallucinations, psychosis;  developed gait instability and disorganized speech after 5 days. |  | WBC: 2  Protein: 33mg/dL  IgG: 38.40 mg/L  OCB: Neg | ED in frontal occipital temporal regions | Normal | S: 1:100  CSF: 1:1 |
| 4  M  13 | Fever,  headache | None | NA | Generalized tonic-clonic seizure;  experienced relapse  with seizure at 9 months. | Oxcarbazepine, clonazepam, lamotrigine;  after relapse,  the regimen was changed to levetiracetam, clonazepam, lacosamide. | WBC: 16  Protein: 22mg/dL  IgG: 17.94 mg/L | Generalized slow waves; ED in  L temporal regions | Normal | S: 1:10  CSF: NA |
| 5  M  69 | Fever,  cough,  sore throat | None | Chest, abdominal  and pelvis CT | Psychomotor slowing, memory loss,  Irritability. |  | WBC: 5  Protein: 64mg/dL  IgG: 38.3mg/L | Abnormal | Normal | S: 1:32  CSF: Neg |
| 6  M  67 | None | None | Chest, abdominal  and pelvis CT,  PET-CT | Memory loss,  psychomotor slowing |  | WBC: 41  Protein: 95mg/dL  IgG: 575 mg/L | Diffused slow waves | Bi hippocampus | S: 1:10  CSF: Neg |
| 7  M  51 | None | None | Serum tumor  markers | Auditory hallucinations, persecutory delusion; psychosis,  agitation,  memory loss and difficulty walking  at 3 months. |  | WBC: 16  Protein: 73mg/dL  IgG: 138 mg/L | NA | Bi  frontal and parietal lobes | S: 1:10  CSF: Neg |
| 8  M  58 | None | None | Chest, abdominal  and pelvis CT, | Personality changes, agitation,  depressed mood, memory loss,  sleep disorder. |  | NA | Slow waves, ED | Unremarkable | S: 1:10  CSF: NA |
| 9  M  32 | Headache,  fever | None | Serum tumor  markers,  PET-CT | Personality changes, irritability,  aggressive behavior, apathy,  auditory hallucinations, memory deficits, attention deficits, insomnia. |  | WBC: Normal Protein:50mg/dL IgG:69.8mg/L  IgG index: Increased | Normal | Normal at onset and  1st relapse;  Bi hippocampus  at 2nd relapse | S: 1:320 CSF: 1:10 |
| 10  M  51 | Fever | None | Serum tumor  markers | Agitation,  partial and generalized seizures,  status epilepticus, confusion,  dLOC,  mania,  sweating,  right facial paralysis. | Multiple AEDs were used in combination; specific agents were not reported. | WBC: 43  Protein:400mg/dL IgG:292mg/L | ED in temporal regions | Normal | S: 1:10 CSF: 1:32 |
| 11  F  12 | None | None | Chest CT,  abdominal ultrasonography,  serum tumor  markers | Gyratory seizures involved into secondary generalized tonic-clonic seizure,  prosopagnosia,  memory loss | Oxcarbazepine, lamotrigine; oxcarbazepine was switched to lacosamide after 3 months. | WBC: Normal  Protein: Normal  IgG:19.5 mg/L  OCB: Pos | Frontal intermittent rhythmic delta activity (FIRDA) | Normal | S:NA CSF: 1:32 |
| 12  F  22 | None | None | Chest and  abdominal CT,  lymph nodes ultrasonography,  PET-CT | Simple partial  seizure occurring during sleep,  sleep disturbance | Levetiracetam | WBC: 2  Protein: Normal  IgG: Normal  OCB: Neg | ED in  frontal regions | Normal | S: 1:32  CSF: 1:1 |
| 13  M  44 | None | None | PET-CT | Headache,  vomiting,  Acute Inflammatory Demyelinating Polyneuropathy (AIDP), multiple cranial neuropathies,  bilateral limb weakness and areflexia |  | WBC: 0  Protein:162.7  mg/dL  OCB: Pos | Normal | Normal | S: 1:30  CSF: 1:10 |
| 14  M  38 | Sore throat,  headache | None | PET-CT | Psychosis,  anxiety,  depression,  difficulty in verbal expression,  memory loss;  2 weeks later,  hallucinations, persecutory delusions, agitation,  aggressive behavior, followed by somnolence, delirium,  dLOC | Valproate | WBC: 47  Protein: Normal  IgG: Normal  OCB: Pos | Diffused slow waves | Unremarkable | S: 1:100  CSF: 1:100 |
| 15  F  61 | None | None | Serum tumor  markers,  PET-CT | Focal motor seizures and clonic seizure | Levetiracetam | WBC: Abnormal Protein: Normal | ED in  R central and parietal regions | R  frontal cortex and subcortical regions | S: Pos  CSF: NA |
| 16  M  29 | Fever,  headache, dizziness, muscle soreness | None | Chest CT,  abdominal ultrasonography | Intracranial infection symptoms (severe headache, neck rigidity); 5 days later, psychomotor agitation, intermittent confusion, hiccups. |  | WBC: 222  Protein: 330.5mg/dL | Slow waves | Splenium of  the corpus callosum | S: 1:10  CSF: 1:10 |
| 17  F  35 | Fever,  flu-like | None | Serum tumor  markers,  PET-CT | Personality changes, depressed mood,  apathy,  decreased verbal output, auditory hallucinations, memory deficits, executive dysfunction, insomnia. |  | WBC: Normal  IgG index: Normal | Normal | Normal | S: 1:10  CSF: Neg |
| 18  M  59 | Diarrhea,  flu-like | None | Serum tumor  markers,  PET-CT | Personality changes, irritability,  aggressive behavior, visual hallucinations, aphasia,  memory deficits,  dLOC,  meningeal irritation, hypoventilation. |  | WBC: Normal  IgG index: Normal  OCB: Neg | Diffused slow waves | Diffuse  dura mater | S: 1:100  CSF: 1:10 |
| 19  M  52 | None | None | NA | Fever,  vomiting,  diarrhea,  generalized tonic-clonic seizures,  sensory aphasia,  dLOC;  relapse at 6 weeks, mental abnormality, decreased verbal output, seizures,  loss of time and place orientation. | NA | WBC: 155 | Slow waves; 2nd relapse,  ED in  L posterior temporal, occipital regions | L  medial temporal lobe; 2nd relapse,  L  temporal, occipital,  and insula lobes | S: 1:10  CSF: 1:100 |
| 20  F  22 | None | None | NA | Insomnia,  short-term memory deficits |  | WBC: 7 | Normal | Bi  basal ganglia insula,  medial temporal lobes | S: 1:32  CSF: 1:10 |
| 21  M  36 | None | None | NA | Mixed aphasia, generalized tonic-clonic seizures. | NA | WBC: 2  OCB: Pos | ED in  Bi frontal regions | Normal | S: 1:10  CSF Neg |
| 22  M  51 | None | None | NA | Personality changes, hallucinations,  delusions,  sleep disorders |  | WBC: 16 | Normal | Bi  medial temporal lobes | S: 1:10  CSF: Neg |
| 23  M  58 | None | None | NA | Short-term memory deficits,  absence seizures.  Seizure frequency decreased after 2 months of treatment. | NA | WBC: 1 | Slow waves,  ED in  L anterior temporal and sphenoidal regions | Bi  hippocampus and  L insula lobe | S: 1:100  CSF: Neg |
| 24  M  58 | None | None | NA | Auditory hallucinations, depression,  apathy,  irritability,  REM sleep behavior disorder and reduced sleep duration,  dizziness,  headache,  weight loss,  constipation,  urinary retention, memory loss,  spatial disorientation |  | WBC: 5  IgG index: Increased  OCB: Neg | NA | Normal | S: +++ CSF: Neg |
| 25  M  30 | Diarrhea | None | NA | Focal seizures with impaired awareness and focal to bilateral tonic-clonic seizures,  memory loss | NA | WBC: 3  IgG index: Normal  OCB: Neg | NA | Normal | S: +  CSF: Neg |
| 26  M  17 | None | None | NA | Memory loss,  focal seizures with impaired awareness | NA | WBC: 1  IgG index: Normal  OCB: Neg | NA | Bi  medial temporal lobes and insula, enlarged  L amygdala | S: +  CSF: Neg |
| 27  F  68 | None | None | NA | Memory loss,  headache with tinnitus, daytime sleepiness,  focal seizures with impaired awareness and focal to bilateral tonic-clonic seizures;  urinary and fecal incontinence | NA | WBC: 2 | NA | Normal | S: +  CSF: Neg |
| 28  F  70 | None | None | NA | Auditory and visual hallucinations, persecutory delusion, mumble to herself, difficulty in falling and sustaining sleep, nightmares,  memory loss |  | WBC: 1  IgG index: Normal  OCB: Neg | NA | Subdural effusion in the R  fronto-temporal region | S: +  CSF: Neg |
| 29  F  22 | None | None | NA | Frequent focal seizures without impaired awareness,  nocturnal awakening, fever | NA | WBC: 2  IgG index: Normal  OCB: Neg | NA | Normal | S: ++  CSF: + |
| 30  F  46 | None | None | NA | Transient numbness and weakness in left lower limb,  then complex partial seizure,  memory loss,  depression | NA | WBC: 1  IgG index:  Normal  OCB: Neg | NA | R hippocampal, medial temporal and white matter | S: +  CSF: Neg |
| 31  F  19 | None | None | NA | Focal seizures with impaired awareness, anxiety,  memory loss,  poor sleep | NA | WBC: 4  OCB: Neg | NA | Normal | S: +  CSF: NA |
| 32  F  38 | Flu-like, diarrhea,  fever | None | NA | Asthenia,  anorexia,  nausea,  vomiting and emotional instability |  | WBC: 9  IgG index: Normal  OCB: Neg | NA | Enhancement in R cerebellar tentorium | S: +  CSF: Neg |
| 33  F  78 | Flu-like | None | NA | Mild dizziness,  walking difficulty due to persist left limb weakness,  somnolence,  memory loss,  depression,  anxiety,  manic,  spatiotemporal disorientation, impairment of verbal comprehension, distension, weight loss, incontinence |  | WBC: 13  IgG index: Normal  OCB: Pos | NA | Ring enhancement of multiple thin-walled well-circumscribed lesions with varying sizes | S: ++  CSF: + |
| 34  M  49 | None | None | NA | Dizziness,  diplopia,  feet numbness,  walking difficulty,  status epilepticus,  words are difficult to understand,  dysphagia,  paroxysmal involuntary movements in upper limbs and mandibular, fever | NA | WBC: 2  IgG index: Normal  OCB: Neg | NA | Subdural effusion in the R  fronto-temporal region | S: +  CSF: Neg |
| 35  M  65 | High fever | None | NA | Slurred speech,  neck stiffness,  vomiting,  somnolence,  mutism,  no reaction to pain in the upper limbs,  generalized seizures | NA | WBC: 29  IgG index: Increased  OCB Neg | NA | Subdural effusion | S: ++  CSF: ++ |
| 36  M  71 | Heat,  pain,  redness and swelling  in the left ear, headache,  fever | None | NA | Auditory and visual hallucinations,  babbling and persecutory delusion, prosopagnosia, memory loss,  focal seizures with impaired consciousness, slow responses; constipation and slight alterations of continence | NA | WBC: 163  IgG index: Increased  OCB: Pos | NA | Bi hippocampi, medial temporal and subcortical matter of frontal lobes | S: ++  CSF: Neg |
| 37  F  7 | Intermittent fever | None | Chest, abdominal  and pelvis CT,  lymph nodes ultrasonography,  PET-CT | Shwachman-Diamond syndrome (SDS), seizures,  behavioral abnormalities (episodes of eye rolling, mouth twitching, pronounced mouth breathing,  followed by sudden rising and engaging in self-talk and hand-and-foot movements), seizures | Levetiracetam | WBC: 1  Protein: 18 mg/dL  IgG: Normal  OCB: Neg | Slow waves, ED | Normal | S: 1:1000  CSF: Neg |
| 38  M  12 | Headache,  fever | Gangliocytoma | Abdominal CT, abdominal  ultrasonography | Irritability,  auditory hallucinations,  sleep disorders,  memory and comprehension decrease, |  | WBC: 90  IgG index: Increased  OCB: Pos | Normal | R insular lobe | S: 1:100  CSF:1:3.2 |
| 39  F  37 | Headache | Pulmonary adenocarcinoma | Chest CT,  lymph nodes and  ultrasonography,  serum tumor  markers,  PET-CT | Dysarthria,  memory deficits, bilateral hearing loss, followed by seizure activity,  dLOC |  | OCB: Neg  WBC: 28 | Normal | L  basal ganglia,  frontal lobe, parietal lobe | S:1:10  CSF: Neg |

Patients 1-8 were from our center.

Isolated anti-mGluR5 antibodies refer to patients with anti-mGluR5 antibody positivity without coexisting antibodies.

Abbreviations: AEDs, antiepileptic drugs; CSF, cerebrospinal fluid; EEG, electroencephalography; MRI, magnetic resonance imaging; Ab, antibody; CT, computed tomography; NA, not available; unperformed in institutional cases or unreported in literature-derived; ED, epileptiform discharge; S, serum; IgG, immunoglobulin; WBC, White Blood Cell per mm3; OCB, oligoclonal bands; Neg, negative; PET-CT, positron emission tomography-computed tomography; L, left; Bi, bilateral; dLOC, decreased level of consciousness; Pos, positive; R, right.

**Table S1B. Chinese patients with multiple antibodies (n=13)**

| ID  Sex  Age | Prodromal Symptoms | Tumor | Tumor Screening  Strategies | Main clinical features | AEDs | CSF Findings | EEG | MRI | Ab Titer /  Co-existing Ab |
| --- | --- | --- | --- | --- | --- | --- | --- | --- | --- |
| 40  M  71 | None | None | Chest, abdominal  and pelvis CT | Depressed mood,  limb tremors,  decreased verbal output, apathy;  developed memory loss and generalized tonic-clonic seizures 4 months later. | Oxcarbazepine | Protein: 88mg/dL  IgG: 88.1mg/L  4 WBC | ED | L frontal lobe,  posterior horn of the lateral ventricle, midbrain | S: 1:10  CSF: 1:1;  LGI1  S: 1:100  CSF: 1:100 |
| 41  M  31 | None | HD | Chest, abdominal  and pelvis CT,  PET-CT | Headache,  vertigo,  diplopia;  experienced projectile vomiting and disorganized speech  2 months later. |  | Protein: 51.8mg/dL  IgG: 37.8mg/L  Normal WBC  OCB: Pos | Normal | Bi cerebellar, frontal and parietal white matter | S: 1:10  CSF: Neg  GAD65  S: 1:10  CSF: 1:1;  Yo  S: 1:10  CSF: Neg |
| 42  F  12 | Fever,  headache,  flu-like | None | Chest, abdominal  and pelvis CT | Headache,  dizziness,  irritability,  agitation,  memory loss,  mental and behavioral disturbances. |  | Protein: 42mg/dL  IgG: 10.3mg/L  1 WBC  OCB: Neg | NA | Normal | S: 1:10  CSF: Neg;  IgLON5  S: 1:10  CSF: Neg |
| 43  M  38 | Fever,  headache | None | Chest and abdominal CT,  serum tumor markers | Difficulty in language expression;  1 week later,  psychomotor agitation, generalized tonic-clonic seizure,  status epilepticus | Oxcarbazepine | Protein:141.7  mg/dL  396 WBC | ED in  L frontal and temporal lobes | Extensive cortical edema in L cerebral hemisphere | S: 1:10  CSF: 1:10;  MOG  S:1:32  CSF: 1:10;  NMDAR  S: Neg  CSF: 1:10 |
| 44  F  65 | None | None | Chest and abdominal CT,  gynecological ultrasonography | Facial-brachial dystonia episodes (FBDS), unresponsive,  seizures,  confusion | Levetiracetam,  valproate | Protein: Normal  Normal WBC | Abnormal | R caudate nucleus and putamen | S: 1:10  CSF: Neg;  LGI1  S: 1:100, CSF: 1:30 |
| 45  M  15 | Fever,  sore throat | None | Chest CT | dLOC,  seizures,  agitation,  aggressive behavior | Multiple AEDs were used in combination; specific agents were not reported | Protein: Normal  Normal WBC | Generalized slow waves | Normal | S: NA  CSF: Pos;  NMDAR  S: NA  CSF: Pos |
| 46  F  26 | None | Ovarian Teratoma | Chest, abdominal  and pelvis CT,  gynecological ultrasonography,  serum tumor markers, | Irritability,  babbling,  stiffness of the limbs, sleepwalking, hallucinations and paroxysmal mania;  3 weeks later, drowsiness,  difficulty with attention.  Symptoms improved significantly after 1 month of treatment. |  | Protein:119.5  mg/dL  IgG: Normal  4 WBC | Diffused slow wave | Splenium of corpus callosum | S: 1:10  CSF: Neg;  NMDAR  S: 1:32  CSF: 1:3.2 |
| 47  M  36 | Headache,  flu-like | None | serum tumor markers,  PET-CT | Personality changes, behavioral changes with irritability,  mania,  visual hallucinations, insomnia,  visual deficits |  | IgG index: Increased  80 WBC  OCB: Neg | Normal | R mesio-temporal lobe,  cerebral peduncle, thalamus and putamen | S: Neg  CSF: 1:10;  Recoverin  S: Pos  CSF: NA |
| 48  F  35 | Headache | Mature teratoma | serum tumor markers,  PET-CT | Spatial disorientation, prosopagnosia,  memory deficits,  visual hallucinations, generalized seizures, status epilepticus,  dLOC,  coma,  dystonia,  hypoventilation. | NA | IgG index: Increased  120 WBC | Focal slow waves, ED | Bi hippocampi | S: 1:10  CSF: 1:100;  NMDAR  S: Pos  CSF: Pos; AMPAR  S: Neg  CSF: Pos |
| 49  F  16 | Headache | None | NA | Generalized tonic-clonic seizure,  followed by weight loss, sleep disturbances, constipation,  irritable,  crying,  anxiety,  memory loss | NA | IgG index:  Normal  3 WBC  OCB: Pos | NA | R  hippocampus | S: +  CSF: Neg  LGI1  S: +  CSF: Neg |
| 50  M  29 | None | None | NA | Focal to bilateral tonic-clonic seizures | NA | 0 WBC | NA | Ischemic foci in white matter | S: +  CSF: Neg;  Amphi-physin  S: +  CSF: Neg |
| 51  F  60 | Headache | None | Chest CT, abdominal ultrasonography,  serum tumor markers | Progressive vision loss |  | Protein: 23 mg/dL  1 WBC | Normal | Posterior horn of the  L lateral ventricle | S:1:32  CSF: Neg;  MOG  S:1:10  CSF: Neg |
| 52  F  21 | None | Teratoma | Pelvic CT | Mental and behavioral disturbances,  limb convulsions,  fever,  dLOC,  seizures | Phenobarbital, valproate, levetiracetam | Protein: 11 mg/dL  127 WBC | Waves and spike rhythms activity in the R frontal region | Bi  hippocampus,  R temporal parietal lobe | S:1:30  CSF:1:30;  NMDAR  S:1:100  CSF:1:100 |

Patients 40-42 were from our center.

Multiple antibodies refer to patients with anti-mGluR5 antibody positivity together with coexisting antibodies.

Abbreviations: AEDs, antiepileptic drugs; CSF, cerebrospinal fluid; EEG, electroencephalography; MRI, magnetic resonance imaging; Ab, antibody; CT, computed tomography; IgG, immunoglobulin; WBC, White Blood Cell per mm3; ED,epileptiform discharge; L, left; S, serum; HD, Hodgkin disease; PET-CT, positron emission tomography-computed tomography; OCB, oligoclonal bands; Pos, positive; Bi, bilateral; Neg, negative; NA, not available; unperformed in institutional cases or unreported in literature-derived; R, right; dLOC decreased level of consciousness.

**Table S1C. Non-Chinese patients (n=14)**

| ID  Sex  Age | Prodromal features | Tumor | Tumor Screening  Strategies | Main clinical features | AEDs | CSF analysis | EEG | MRI | Ab titer |
| --- | --- | --- | --- | --- | --- | --- | --- | --- | --- |
| 53  F  46 | None | HD, stage 3A | Chest CT | Personality changes and depression for 1 year, then seizures,  memory loss,  emotional lability, myoclonic jerks,  and tremor | NA | WBC: 23  Protein: 55mg/dL | NA | At onset: unilateral  R mesio-temporal lobe.  At 3 months: Bi temporal, thalamus, insula, frontal. Gd+ | S: +  CSF: NA |
| 54  M  15 | Headache, nausea | HD, stage2A | PET-CT | Confusion,  auditory and visual hallucinations,  decreased verbal output, attention deficit,  status epilepticus | NA | WBC: 114  Protein: 40mg/dL  OCB: Pos | NA | Bi (L > R),  posterior cortical diffusion restriction | S: NA  CSF: +++ |
| 55  M  35 | Weight loss | HD, stage 2B | Chest CT | Aggressive behavior, depressed mood and anxiety,  memory loss,  right X, XI, XII nerve palsy |  | WBC: 12  Protein: 87mg/dL  IgG index: Increased  OCB: Neg | Abnormal | Bi (R> L),  upper pons. Gd+ | S: NA  CSF: 1:160 |
| 56  F  30 | Weight loss, flu-like | None | PET-CT | Personality changes, aggressive behavior, hypersomnia,  memory loss, visuospatial deficit, prosopagnosia,  dLOC,  seizures;  relapse at 16 months | Levetiracetam | WBC: 25  IgG Index: Increased  OCB: Pos | Abnormal | Normal | S: 1:1280  CSF: 1:320 |
| 57  F  40 | Headache | None | Abdominal CT | Insomnia,  anxiety,  psychosis,  auditory hallucinations, memory loss,  dLOC,  akinetic mutism, orofacial dyskinesia |  | WBC: 45 | NA | Normal | S>>1:1280 CSF: NA |
| 58  M  16 | Headache | HD, stage 3B | NA | Psychosis, hallucinations,  poor sleep,  dystonia,  generalized seizures, dLOC;  neurologic relapse followed by tumor relapse | NA | WBC: 31  OCB: Pos | NA | Normal | S>>1:1280 CSF: 1:20 |
| 59  F  6 | Rash, headache, flu-like | None | NA | Status epilepticus, dLOC, aphasia,  memory loss,  poor sleep with altered sleep-wake cycle, followed by dystonia and oculogyric crisis, psychomotor slowness, ataxia,  speech and motor regression, hypoventilation | NA | WBC: 21  OCB: Neg | NA | Bi frontal (L > R),  R occipital lobes, cerebellum | S: NA  CSF: 1:10 |
| 60  F  20 | Headache, flu-like | None | NA | Psychosis,  emotional lability, thought disorder, anterograde amnesia, psychomotor slowing, hypersomnia |  | WBC: 27  OCB: Pos | NA | Normal | S>>1:1280 CSF: NA |
| 61  M  15 | None | HD, stage 1 | NA | Facial paralysis,  then developed altered behavior,  memory loss,  anxiety,  irritability,  visual hallucinations, insomnia |  | WBC: 45  OCB: Pos | NA | Normal | S: 1:1280  CSF: 1:640 |
| 62  M  49 | None | None | NA | Insomnia,  altered behavior,  mania,  emotional lability, psychomotor agitation, dLOC,  seizures | NA | WBC: 75  OCB: Pos | NA | Normal | S: 1:320  CSF: 1:160 |
| 63  M  68 | Night sweats,  low grade fever,  weight loss | HD, nodular sclerotic variety | NA | Disorientation, inattention,  psychomotor agitation, confusion,  delusional ideas of grandiosity,  auditory hallucinations, and alterations of anterograde memory |  | WBC: Normal  Protein: Normal  OCB: Pos | Normal | Unremarkable | S: NA  CSF: Pos |
| 64  F  30 | None | HD | Whole-body CT | Amnesia,  mutism and nystagmus |  | WBC: 80  Protein: 41mg/dL | None | Bi hippocampus  and mesial temporal lobe ,  internal capsules,  caudate nucleus,  fronto-parietal-occipital lobes,  periaqueductal grey matter | S: Pos  CSF: Pos |
| 65  M  69 | Headache,  flu-like,  weight loss | Acinar adenocarcinoma | Chest, abdominal  and pelvis CT,  serum tumor marker,  PET-CT,  colonoscopy,  prostate biopsy | Jerking movements,  dysarthria,  feeding difficulties, balance issues,  weight loss,  anterograde memory difficulties, prosopagnosia,  visual hallucinations, hypersomnia, snoring, sleep apnea,  seemingly purposeful movements during sleep,  maculopapular rash.  attention and delayed memory deficit, dysarthria, hypophonia, downward vertical gaze palsy,  and gaze fixation impairment,myoclonus,  lower limb pallhypesthesia,  ataxia | Levetiracetam,  valproate,  diazepam |  | Normal | Normal | S:1:10  CSF: Neg |
| 66^a^  M  75 | Weight loss | SCLC | NA | Progressive ophthalmoplegia, postural hand tremor, gait instability,  executive dysfunction |  | WBC: 6  IgG index: Increased | NA | Bi mesio-temporal lobes | S: 1:160 CSF: 1:320 |

**a** Patient 66 had coexisting SOX antibody.

Abbreviations: AEDs, antiepileptic drugs; CSF, cerebrospinal fluid; EEG, electroencephalography; MRI, magnetic resonance imaging; Ab, antibody; HD, Hodgkin disease; CT, computed tomography; WBC, White Blood Cell per mm3; NA, not available; unperformed in institutional cases or unreported in literature-derived; R, right; Bi, bilateral; Gd+, gadolinium enhancement; S, serum; PET-CT, positron emission tomography-computed tomography; OCB, oligoclonal bands; Pos, positive; L, left; IgG, immunoglobulin; Neg, negative; dLOC, decreased level of consciousness.

**Table S2. Treatment strategies and clinical outcomes**

**Table S2A. Chinese patients with isolated anti-mGluR5 antibodies (n=39)**

| ID | Treatment | mRS initial | mRS Last | Onset to immunotherapy interval (weeks) | Follow-up (months) |
| --- | --- | --- | --- | --- | --- |
| 1 | Steroids, IVIg | 2 | 0 | 5 | 35 |
| 2 | Steroids, IVIg | 4 | 0 | 3 | 42 |
| 3 | Steroids, IVIg | 2 | 2 | 10 | 6 |
| 4 | Steroids, IVIg | 5 | 1 | 1 | 48 |
| 5 | Steroids, IVIg | 2 | 0 | 4 | 38 |
| 6 | IVIg | 3 | 2 | 4 | 4 |
| 7 | Steroids | 4 | 2 | 12 | 34 |
| 8^a^ | None | 3 | 1 | NA | 14 |
| 9 | Steroids, IVIg, MMF | 3 | 1 | 3 | 42 |
| 10 | Steroids, IVIg, RTX | 5 | 6 | 7 | 0 |
| 11 | Steroids, IVIg | 3 | 1 | 4 | 3 |
| 12 | IVIg, AZA | 2 | 1 | 9 | 6 |
| 13 | IVIg | 4 | 0 | 1 | 6 |
| 14 | Steroids | 4 | 0 | 3 | 2 |
| 15 | Steroids | 2 | 1 | 17 | 4 |
| 16 | Steroids, IVIg | 3 | 0 | 2 | 6 |
| 17 | Steroids | 3 | 0 | 1 | 18 |
| 18 | Steroids | 5 | 4 | 2 | 11 |
| 19 | Steroids, IVIg | 5 | 4 | NA | 1 |
| 20 | Steroids | 3 | 1 | 5 | 5 |
| 21 | Steroids, IVIg | 3 | 1 | 13 | 6 |
| 22 | Steroids | 3 | 1 | 49 | 4 |
| 23 | Steroids | 2 | 1 | 49 | 2 |
| 24 | Steroids | 3 | 0 | NA | 18 |
| 25 | None | 1 | 0 | NA | 12 |
| 26 | IVIg | 2 | 0 | NA | 12 |
| 27 | Steroids, IVIg | 5 | 1 | NA | 12 |
| 28 | Steroids, IVIg | 3 | 0 | NA | 12 |
| 29 | IVIg, AZA | 2 | 1 | NA | 15 |
| 30 | Steroids | 2 | 0 | NA | 12 |
| 31 | Steroids, PP, MMF | 2 | 1 | NA | 81 |
| 32 | None | 1 | 0 | NA | 58 |
| 33 | Steroids, IVIg | 5 | 3 | NA | 15 |
| 34 | Steroids, IVIg | 5 | NA | NA | NA |
| 35 | None | 5 | 0 | NA | 12 |
| 36 | None | 3 | 0 | NA | 12 |
| 37 | Steroids, IVIg | 3 | 0 | NA | 17 |
| 38 | Steroids, IVIg | 3 | 1 | NA | 1 |
| 39 | IVIg | 1 | 0 | 6 | 2 |

Patients 1-8 were from our center.

Isolated anti-mGluR5 antibodies refer to patients with anti-mGluR5 antibody positivity without coexisting antibodies.

**a** Patient 8 declined any immunotherapy during hospitalization.

Abbreviations: mRS, Modified Rankin scale; IVIg, Intravenous immunoglobulin; NA, not available; for institutional cases, the only missing value was the onset to immunotherapy interval in one untreated patient; for literature-derived cases, missing values indicate unreported data; MMF, mycophenolate mofetil; RTX, rituximab; AZA, azathioprine; PP, plasmapheresis.

**Table S2B. Chinese patients with multiple antibodies (n=13)**

| ID | Treatment | mRS initial | mRS Last | Onset to immunotherapy interval (weeks) | Follow-up (months) |
| --- | --- | --- | --- | --- | --- |
| 40 | Steroids, IVIg | 3 | 1 | 21 | 37  3  171 |
| 41 | Steroids, IVIg, Chemotherapy | 4 | 2 | 4 | 3 |
| 42 | Steroids | 2 | 1 | 3 | 17 |
| 43 | Steroids, IVIg | 3 | 1 | 3 | 6 |
| 44 | Steroids, IVIg | 3 | 0 | NA | 3 |
| 45 | Steroids, IVIg, RTX | 5 | 3 | 3 | 1 |
| 46 | Steroids, IVIg, tumor resection | 3 | 0 | 4 | 9 |
| 47 | Steroids, IVIg | 2 | 0 | 10 | 24 |
| 48 | Steroids, IVIg, tumor resection | 5 | 6 | 2 | 6 |
| 49 | Steroids, IVIg, MMF | 2 | 0 | NA | 18 |
| 50 | Steroids | 1 | 0 | NA | 15 |
| 51 | Steroids, MMF | 3 | 0 | 1 | 1 |
| 52 | Steroids, IVIg | 5 | 6 | 5 | NA |

Patients 40-42 were from our center.

Multiple antibodies refer to patients with anti-mGluR5 antibody positivity together with coexisting antibodies.

Abbreviations: mRS, Modified Rankin scale; IVIg, Intravenous immunoglobulin; NA, not available, indicate unreported data; RTX, rituximab; MMF, mycophenolate mofetil.

**Table S2C. Non-Chinese patients (n=14)**

| ID | Treatment | mRS initial | mRS Last | Onset to immunotherapy interval (weeks) | Follow-up (months) |
| --- | --- | --- | --- | --- | --- |
| 53 | Steroids, ABVD | 3 | 0 | 8 | 48 |
| 54 | Chemotherapy, RT | 5 | 0 | NA | 72 |
| 55 | ABVD | 3 | 0 | NA | 38 |
| 56 | Steroids, PE, RTX | 3 | 1 | NA | 48 |
| 57 | Steroids, PE | 5 | 0 | NA | 20 |
| 58 | Steroids, Chemotherapy, PE | 4 | 0 | NA | 48 |
| 59 | Steroids, IVIg, RTX | 5 | 3 | NA | 19 |
| 60 | None | 4 | 0 | NA | 96 |
| 61 | Steroids, IVIg, Chemotherapy | 4 | 2 | NA | 12 |
| 62 | Steroids | 4 | 1 | NA | 5 |
| 63 | Steroids | 3 | 1 | 24 | 1 |
| 64 | Steroids, IVIg | 3 | 2 | NA | 6 |
| 65 | Steroids, IVIg, PP | 4 | 2 | 12 | 9 |
| 66 | Steroids, IVIg, Chemotherapy, RT | 4 | 3 | NA | 62 |

Abbreviations: mRS, Modified Rankin scale; ABVD, chemotherapy with doxorubicin, bleomycin, vinblastine, and dacarbazine; RT, radiotherapy; NA, not available, indicate unreported data; PE, plasma exchange; RTX, rituximab; IVIg, Intravenous immunoglobulin; PP, plasmapheresis.

**Table S3. Associations of initial clinical symptoms with age, tumor status, antibody-positive site and serum antibody titer in Chinese patients with isolated anti-mGluR5 antibodies**

| **Variable** | **Mental and**  **behavior disorder** | **P value** | **Altered cognition** | **P value** | **Sleep disturbances** | **P value** | **Seizures** | **P value** | **dLOC** | **P value** |
| --- | --- | --- | --- | --- | --- | --- | --- | --- | --- | --- |
| Age |  | 1.000 |  | 1.000 |  | 1.000 |  | 0.190 |  | 0.564 |
| ≤ 20 years | 3/6 (50.0%) |  | 4/6 (66.7%) |  | 2/6 (33.3%) |  | 5/6 (83.3%) |  | 0/6 (0.0%) |  |
| > 20 years | 18/33 (54.5%) |  | 20/33 (60.6%) |  | 12/33 (36.4%) |  | 16/33 (48.5%) |  | 6/33 (18.2%) |  |
| Tumor status |  | 0.586 |  | 1.000 |  | 1.000 |  | 1.000 |  | 0.403 |
| non-tumor | 20/36 (55.6%) |  | 22/36 (61.1%) |  | 13/36 (36.1%) |  | 19/36 (52.8%) |  | 5/36 (13.9%) |  |
| tumor | 1/3 (33.3%) |  | 2/3 (66.7%) |  | 1/3 (33.3%) |  | 2/3 (66.7%) |  | 1/3 (33.3%) |  |
| Antibody positive site |  | 0.966 |  | 0.162 |  | 0.162 |  | 0.579 |  | 0.062 |
| both positive | 8/14 (57.1%) |  | 7/14 (50.0%) |  | 7/14 (50.0%) |  | 6/14 (42.9%) |  | 5/14 (35.7%) |  |
| serum only | 11/19 (57.9%) |  | 14/19 (73.7%) |  | 5/19 (26.3%) |  | 10/19 (52.6%) |  | 1/19 (5.3%) |  |
| Antibody titer |  | 0.675 |  | 0.649 |  | 1.000 |  | 1.000 |  | 0.720 |
| low titer | 9/17 (52.9%) |  | 8/17 (47.1%) |  | 5/17 (29.4%) |  | 7/17 (41.2%) |  | 3/17 (17.6%) |  |
| medium titer | 3/5 (60.0%) |  | 4/5 (80.0%) |  | 1/5 (20.0%) |  | 2/5 (40.0%) |  | 2/5 (40.0%) |  |
| high titer | 2/2 (100.0%) |  | 1/2 (50.0%) |  | 1/2 (50.0%) |  | 1/2 (50.0%) |  | 0/2 (0.0%) |  |

Isolated anti-mGluR5 antibodies refer to patients with anti-mGluR5 antibody positivity without coexisting antibodies.

Only patients with available and analyzable serum antibody titer data were included in the antibody titer analysis (n = 24); cases with missing titer data or semi-quantitative results not reported as specific dilution titers were excluded. Antibody titers were categorized as low (≤ 1:32), medium (>1:32 to <1:320), and high (≥1:320).

Abbreviations: dLOC, decreased level of consciousness.

**Table S4. Association of serum anti-mGluR5 antibody titer with disease severity and prognosis in Chinese patients with isolated anti-mGluR5 antibodies (n=24)**

| **Variable** | **Category** | **Low titer** | **Medium titer** | **High titer** | **P value** |
| --- | --- | --- | --- | --- | --- |
| Disease severity | Initial mRS ≤ 2 | 4/17 (23.5%) | 2/5 (40.0%) | 0/2 (0.0%) | 0.770 |
|  | Initial mRS > 2 | 13/17 (76.5%) | 3/5 (60.0%) | 2/2 (100.0%) |  |
| Prognosis | Follow-up mRS ≤ 2 | 15/17 (88.2%) | 4/5 (80.0%) | 2/2 (100.0%) | 1.000 |
|  | Follow-up mRS > 2 | 2/17 (11.8%) | 1/5 (20.0%) | 0/2 (0.0%) |  |

Isolated anti-mGluR5 antibodies refer to patients with anti-mGluR5 antibody positivity without coexisting antibodies.

Only patients with analyzable serum antibody titer data were included (n = 24); cases with missing titer data or semi-quantitative results not reported as specific dilution titers were excluded. Disease severity and prognosis were represented by the initial and follow-up mRS scores, respectively. Antibody titers were categorized as low (≤ 1:32), medium (>1:32 to <1:320), and high (≥1:320).

Abbreviations: mRS, Modified Rankin Scale.

**Table S5. Comparison of treatment regimens and outcomes in Chinese patients with isolated anti-mGluR5 antibodies (n=33)**

| **Treatment regimen** | **Favorable Prognosis** | **Unfavorable Prognosis** | **P value** |
| --- | --- | --- | --- |
| IVIg + Steroids | 13/16 (81.2%) | 3/16 (18.8%) | 0.645 |
| IVIg alone | 6/6 (100.0%) | 0/6 (0.0%) |  |
| Steroids alone | 10/11 (90.9%) | 1/11 (9.1%) |  |

Isolated anti-mGluR5 antibodies refer to patients with anti-mGluR5 antibody positivity without coexisting antibodies.

Abbreviations: IVIg, Intravenous immunoglobulin.

**Table S6. Quality assessment of included studies**

**Table S6A. Quality assessment for the included case report studies**

| **Articles** | **D1** | **D2** | **D3** | **D4** | **D5** | **D6** | **D7** | **D8** | | **Overall Appraisal**  **(Score)** |
| --- | --- | --- | --- | --- | --- | --- | --- | --- | --- | --- |
| Mat A et al. (2013) | Yes | Yes | Yes | Yes | Yes | Yes | Unclear | Yes | | 7/8 |
| Prüss H et al. (2014) | Yes | Yes | Yes | Yes | Yes | Yes | Unclear | Yes | | 7/8 |
| Guevara C et al. (2018) | Yes | Yes | Yes | Yes | Yes | Yes | Unclear | Yes | | 7/8 |
| Yingbei L et al. (2021) | Yes | Yes | Yes | Yes | Yes | Yes | Yes | Yes | | 8/8 |
| Yueqiu C et al. (2021) | Yes | Yes | Yes | Yes | Yes | Yes | Yes | Yes | | 8/8 |
| Kundian G et al. (2021) | Yes | Yes | Yes | Yes | Yes | Yes | Unclear | Yes | | 7/8 |
| Chenglong M et al. (2022) | Yes | Yes | Yes | Yes | Yes | Yes | Unclear | Yes | | 7/8 |
| Yu J et al. (2022) | Yes | Yes | Yes | Yes | Yes | Yes | Yes | Yes | | 8/8 |
| Tiantian H et al. (2022) | Yes | Yes | Yes | Yes | Yes | Yes | Yes | Yes | | 8/8 |
| Weiqian Y et al. (2022) | Yes | Yes | Yes | Yes | Yes | Yes | Unclear | Yes | | 7/8 |
| Jie F et al. (2022) | Yes | Yes | Yes | Yes | Yes | Yes | Unclear | Yes | | 7/8 |
| Mingmin Z et al. (2023) | Yes | Yes | Yes | Yes | Yes | Yes | Unclear | Yes | | 7/8 |
| Yaqiang L et al. (2023) | Yes | Yes | Yes | Yes | Yes | Yes | Yes | Yes | | 8/8 |
| Yu Z et al. (2023) | Yes | Yes | Yes | Yes | Yes | Yes | Yes | Yes | | 8/8 |
| Pedrosa DA et al. (2024) | Yes | Yes | Yes | Yes | Yes | Yes | Unclear | Yes | | 7/8 |
| Salles PA et al. (2024) | Yes | Yes | Yes | Yes | Yes | Yes | Yes | Yes | | 8/8 |
| Kaili S et al. (2024) | Yes | Yes | Yes | Yes | Yes | Yes | Yes | Yes | | 8/8 |
| Jianhang H et al. (2024) | Yes | Yes | Yes | Yes | Yes | Yes | Unclear | Yes | | 7/8 |
| Xiaoling Z et al. (2024) | Yes | Yes | Yes | Yes | Yes | Yes | Unclear | Yes | | 7/8 |
| Yixin Gu, et al. (2025) | Yes | Yes | Yes | Yes | Yes | Yes | Yes | Yes | | 8/8 |
| **D1** | Were patient's demographic characteristics clearly described? | | | | | | | |  | |
| **D2** | Was the patient's history clearly described and presented as a timeline? | | | | | | | |  | |
| **D3** | Was the current clinical condition of the patient on presentation clearly described? | | | | | | | |  | |
| **D4** | Were diagnostic tests or assessment methods and the results clearly described? | | | | | | | |  | |
| **D5** | Was the intervention(s) or treatment procedure(s) clearly described? | | | | | | | |  | |
| **D6** | Was the post-intervention clinical condition clearly described? | | | | | | | |  | |
| **D7** | Were adverse events (harms) or unanticipated events identified and described? | | | | | | | |  | |
| **D8** | Does the case report provide takeaway lessons? | | | | | | | |  | |

**Table S6B. Quality assessment for the included case series studies**

| **Articles** | **D1** | **D2** | **D3** | **D4** | **D5** | **D6** | **D7** | **D8** | **D9** | **D10** | **Overall Appraisal**  **(Score)** |
| --- | --- | --- | --- | --- | --- | --- | --- | --- | --- | --- | --- |
| Lancaster E et al. (2011) | Unclear | Yes | Yes | Unclear | Unclear | Yes | Yes | Yes | Yes | No | 6/10  (multiple case reports) |
| Spatola M et al. (2018) | Yes | Yes | Yes | Unclear | Unclear | Yes | Yes | Yes | Yes | Yes | 8/10 |
| Guomin X et al. (2022) | Unclear | Yes | Yes | Unclear | Unclear | Yes | Yes | Yes | Yes | No | 6/10  (multiple case reports) |
| Jilun F et al. (2022) | Yes | Yes | Yes | Unclear | Yes | Yes | Yes | Yes | Yes | Yes | 9/10 |
| Sixian C et al. (2023) | Yes | Yes | Yes | Unclear | Unclear | Yes | Yes | Yes | Yes | No | 7/10 |
| Kundian G et al. (2023) | Yes | Yes | Yes | Unclear | Unclear | Yes | Yes | Yes | Yes | Yes | 8/10 |
| Yueqian S et al. (2023) | Yes | Yes | Yes | Unclear | Unclear | Yes | Yes | Yes | Yes | Yes | 8/10 |
| **D1** | Were there clear criteria for inclusion in the case series? | | | | | | | | | |  |
| **D2** | Was the condition measured in a standard, reliable way for all participants included? | | | | | | | | | |  |
| **D3** | Were valid methods used for identification of the condition for all participants included? | | | | | | | | | |  |
| **D4** | Did the case series have consecutive inclusion of participants? | | | | | | | | | |  |
| **D5** | Did the case series have complete inclusion of participants? | | | | | | | | | |  |
| **D6** | Was there clear reporting of the demographics of the participants in the study? | | | | | | | | | |  |
| **D7** | Was there clear reporting of clinical information of the participants? | | | | | | | | | |  |
| **D8** | Were the outcomes or follow-up results of cases clearly reported? | | | | | | | | | |  |
| **D9** | Was there clear reporting of the presenting site(s)/clinic(s) demographic information? | | | | | | | | | |  |
| **D10** | Was statistical analysis appropriate? | | | | | | | | | |  |


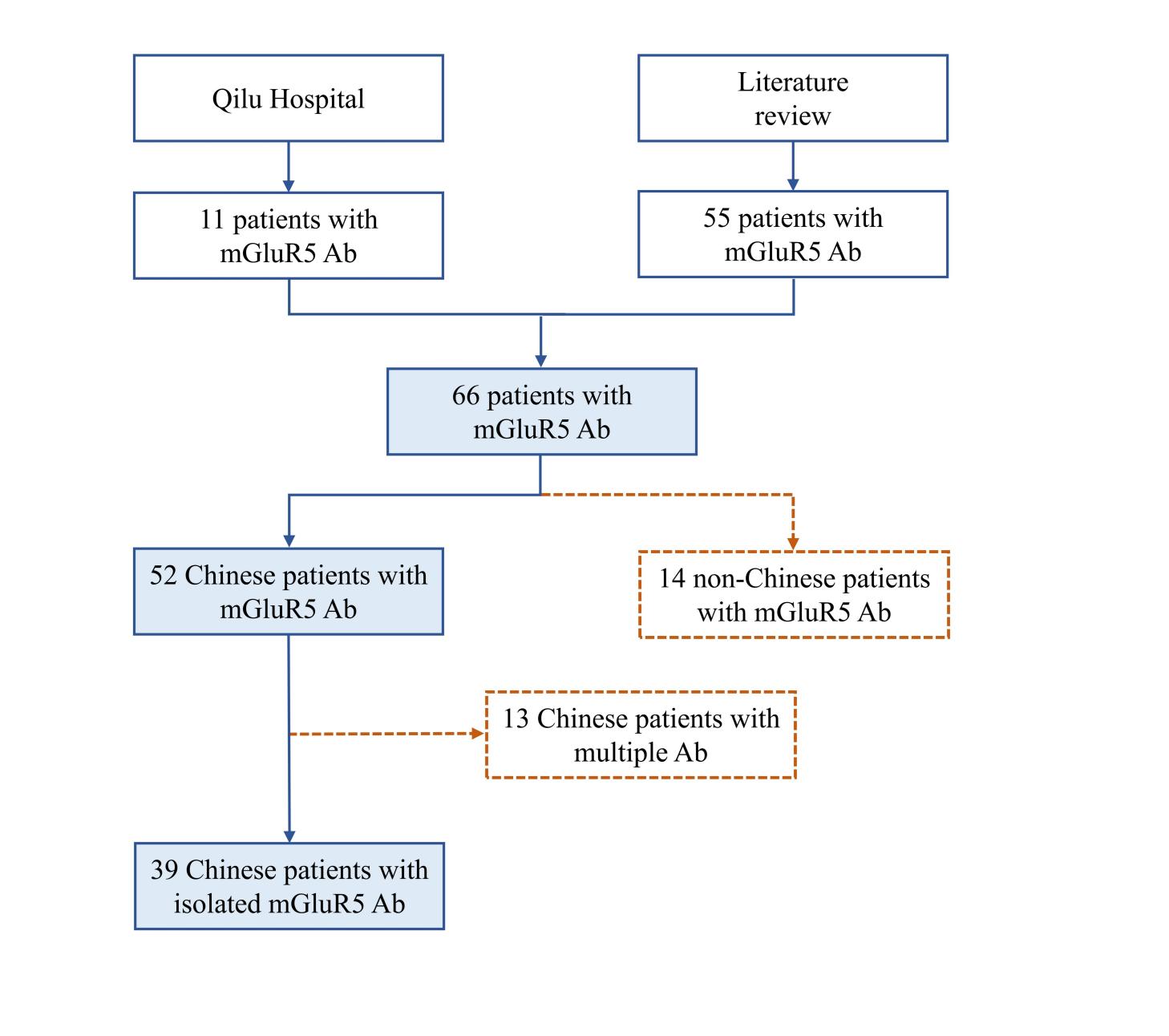


**Supplemental Figure S1. Screening and grouping process for patients with mGluR5 antibodies.** Among the 14 non-Chinese patients, 13 had isolated antibodies and 1 had multiple antibodies. Isolated anti-mGluR5 antibodies refer to patients with anti-mGluR5 antibody positivity without coexisting antibodies. Multiple antibodies refer to patients with anti-mGluR5 antibody positivity together with coexisting antibodies.

Abbreviations: Ab, antibody.

**
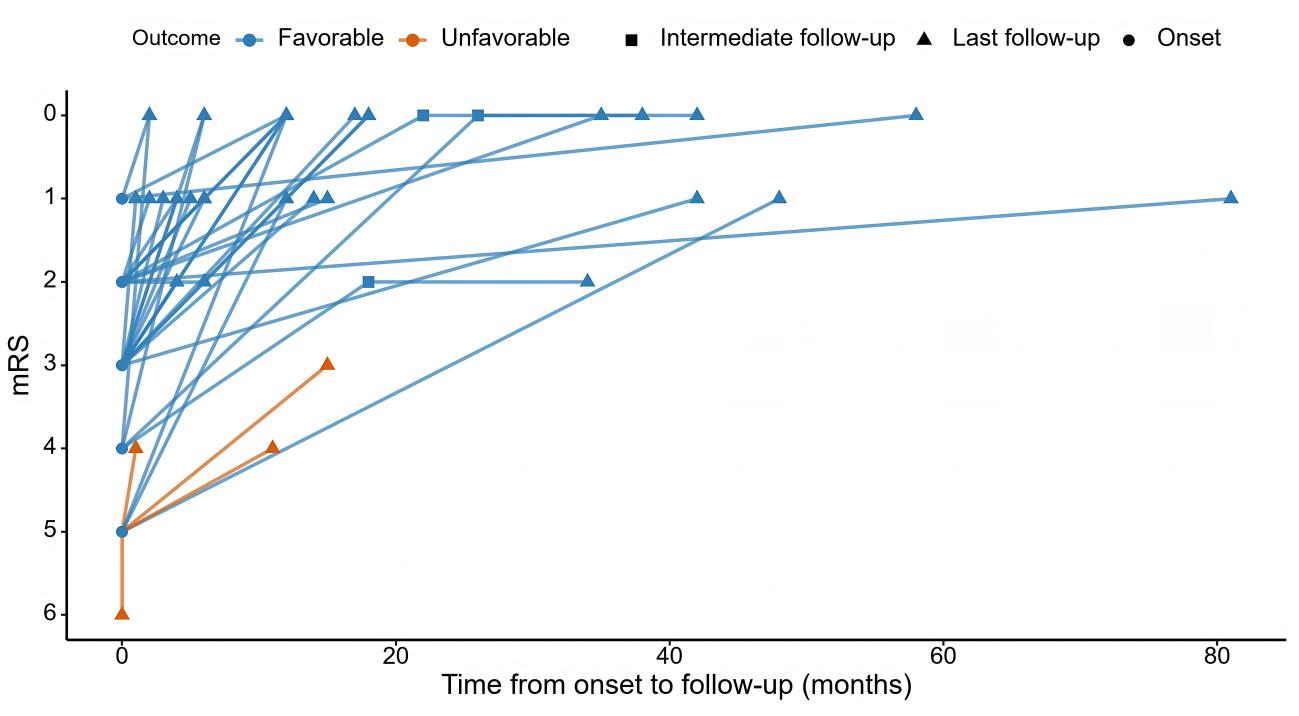
Supplemental Figure S2. Longitudinal follow-up trajectories of mRS scores in Chinese patients with isolated anti-mGluR5 encephalitis.** A total of 38 patients had available follow-up data. Among them, four institutional patients underwent an additional intermediate follow-up assessment and therefore had 3 recorded time points.

Abbreviations: mRS, Modified Rankin Scale.
